# A Surface Exposed, Two-Domain Lipoprotein Cargo of a Type XI Secretion System Promotes Colonization of Host Intestinal Epithelia Expressing Glycans

**DOI:** 10.3389/fmicb.2022.800366

**Published:** 2022-04-29

**Authors:** Alex S. Grossman, Cristian A. Escobar, Erin J. Mans, Nicholas C. Mucci, Terra J. Mauer, Katarina A. Jones, Cameron C. Moore, Paul E. Abraham, Robert L. Hettich, Liesel Schneider, Shawn R. Campagna, Katrina T. Forest, Heidi Goodrich-Blair

**Affiliations:** ^1^Department of Microbiology, The University of Tennessee, Knoxville, Knoxville, TN, United States; ^2^Department of Bacteriology, The University of Wisconsin–Madison, Madison, WI, United States; ^3^Department of Chemistry, The University of Tennessee, Knoxville, Knoxville, TN, United States; ^4^Biosciences Division, Oak Ridge National Laboratory, Oak Ridge, TN, United States; ^5^Department of Animal Sciences, The University of Tennessee, Knoxville, Knoxville, TN, United States; ^6^Biological and Small Molecule Mass Spectrometry Core, The University of Tennessee, Knoxville, Knoxville, TN, United States; ^7^The University of Tennessee Oak Ridge Innovation Institute, Knoxville, TN, United States

**Keywords:** type XI secretion, symbiosis, Slam, lipidation, proteomics, metabolomics, NMR

## Abstract

The only known required component of the newly described Type XI secretion system (TXISS) is an outer membrane protein (OMP) of the DUF560 family. TXISS_OMPs_ are broadly distributed across proteobacteria, but properties of the cargo proteins they secrete are largely unexplored. We report biophysical, histochemical, and phenotypic evidence that *Xenorhabdus nematophila* NilC is surface exposed. Biophysical data and structure predictions indicate that NilC is a two-domain protein with a C-terminal, 8-stranded β-barrel. This structure has been noted as a common feature of TXISS effectors and may be important for interactions with the TXISS_OMP_. The NilC N-terminal domain is more enigmatic, but our results indicate it is ordered and forms a β-sheet structure, and bioinformatics suggest structural similarities to carbohydrate-binding proteins. *X. nematophila* NilC and its presumptive TXISS_OMP_ partner NilB are required for colonizing the anterior intestine of *Steinernema carpocapsae* nematodes: the receptacle of free-living, infective juveniles and the anterior intestinal cecum (AIC) in juveniles and adults. We show that, in adult nematodes, the AIC expresses a Wheat Germ Agglutinin (WGA)-reactive material, indicating the presence of *N*-acetylglucosamine or *N*-acetylneuraminic acid sugars on the AIC surface. A role for this material in colonization is supported by the fact that exogenous addition of WGA can inhibit AIC colonization by *X. nematophila*. Conversely, the addition of exogenous purified NilC increases the frequency with which *X. nematophila* is observed at the AIC, demonstrating that abundant extracellular NilC can enhance colonization. NilC may facilitate *X. nematophila* adherence to the nematode intestinal surface by binding to host glycans, it might support *X. nematophila* nutrition by cleaving sugars from the host surface, or it might help protect *X. nematophila* from nematode host immunity. Proteomic and metabolomic analyses of wild type *X. nematophila* compared to those lacking *nilB* and *nilC* revealed differences in cell wall and secreted polysaccharide metabolic pathways. Additionally, purified NilC is capable of binding peptidoglycan, suggesting that periplasmic NilC may interact with the bacterial cell wall. Overall, these findings support a model that NilB-regulated surface exposure of NilC mediates interactions between *X. nematophila* and host surface glycans during colonization. This is a previously unknown function for a TXISS.

## Introduction

Bacteria rely on secretion systems to convey proteins across membranes to the cell surface and extracellular environment. In host-associated bacteria, such effector proteins, which can include surface-exposed lipoproteins, mediate acquisition of nutrients, signaling interactions with host cells, mechanical interactions with host surfaces, and specificity in host range. These processes make effector proteins potential targets for pharmaceutical treatment and vaccine development (Konovalova and Silhavy, 2015; Wilson and Bernstein, 2016; Kinkead et al., 2018). Bacterial lipoproteins are classified by N-terminal lipidation at a cysteine residue but, otherwise, are diverse in sequence, function, and subcellular localization. The mechanisms by which certain classes of proteins, including lipoproteins, are targeted to and oriented within the outer-membrane are still largely unknown. The newly described type XI secretion system (TXISS), comprising an outer membrane protein (OMP) containing a DUF560 (a domain of unknown function 560), is broadly distributed among proteobacteria and mediates translocation of lipoprotein and a soluble protein cargo across the outer membrane (Heungens et al., 2002; Bhasin et al., 2012; Hooda et al., 2017; Grossman et al., 2021).

A sequence-similarity-based network analysis provided functionally relevant categorization of these TXISS OMPs, hereafter referred to as TXISS_OMPs_, into 10 clusters. Cluster 1, which contains the largest number of TXISS_OMPs_, was further refined into three subclusters: A, B, and C. Clusters 1A and 1B contain the few characterized TXISS OMPs, and its members predominantly are encoded by microbes isolated from animals (Grossman et al., 2021). *Neisseria meningitidis* Slam1 and Slam2 are TXISS_OMPs_ responsible for secretion of transferrin-, lactoferrin-, factor H- or hemoglobin/haptoglobin-binding lipoproteins. *X. nematophila* HrpB and *Acinetobacter baumanii* HsmA are TXISS_OMPs_ that secrete the soluble (non-lipidated) hemophores HrpC and HrpA, respectively. *Xenorhabdus nematophila* NilB is a TXISS_OMP_ that, along with the associated lipoprotein NilC, is necessary for mutualistic colonization of the nematode *Steinernema carpocapsae* (Cowles and Goodrich-Blair, 2008; Hooda et al., 2016, 2017; Fantappie et al., 2017; da Silva et al., 2019; Bateman et al., 2021; Grossman et al., 2021).

Current evidence indicates TXISS_OMPs_ have specificity for their cargo. When expressed in *Escherichia coli* BL21-C43, *Neisseria* TXISS-1A homologs Slam1 and Slam2 do not translocate each other’s cargo proteins, nor can Slam1 translocate the *E. coli* periplasmic lipoprotein PgaB (Hooda et al., 2016). TXISS cargo proteins for which there are known structures (TbpB, LbpB, fHbp, HupA) have limited sequence similarity but a common organization of an N-terminal effector domain and a C-terminal 8-stranded β-barrel that may direct a cargo for secretion (Hooda et al., 2017). The TbpB C-terminal β-barrel is the first part of the cargo protein to be surface exposed during secretion (Hooda et al., 2016), which may indicate this domain initiates interactions with TXISS_OMPs_. Hooda et al. (2017) suggested that, in contrast to the common C-terminal β-barrel, the N-terminal regions of TXISS cargo proteins are variable. This suggests a general conceptual framework in which the cargo C-terminus is necessary for its interaction with a TXISS_OMP_, while the N-terminus encodes the host interaction (or other) effector domain.

An *X. nematophila* TXISS_OMP_, NilB, is encoded near an outer membrane lipoprotein NilC on a locus known as Symbiosis Region 1 (SR1) (Heungens et al., 2002; Cowles and Goodrich-Blair, 2004; Bhasin et al., 2012). In *X. nematophila*, the SR1 locus, which encodes both *nilB* and *nilC*, is necessary and sufficient for normal levels of colonization of *S. carpocapsae* intestines (Heungens et al., 2002; Cowles and Goodrich-Blair, 2008; Chaston et al., 2013). *nilB* and *nilC* are not organized in an operon and have two different promoter sequences, but they are coordinately downregulated at the transcriptional level by the transcription factors NilR and Lrp in a synergistic manner (Cowles and Goodrich-Blair, 2006). Although NilB and NilC function in *S. carpocapsae* colonization is well-established, their cellular and molecular functions remain unclear. Given that NilB is a member of the TXISS_OMP_ family that facilitates the surface exposure of target lipoproteins, we considered the model that it functions to facilitate NilC surface exposure, and that NilC is a host-interaction effector. We describe here experiments to determine if NilB and NilC form a TXISS_OMP_–host interaction effector pair by determining if NilC lipoprotein can be surface exposed, if NilB facilitates NilC lipoprotein surface exposure, and what effector activity one or both of them might have with respect to host interactions.

## Results

### NilB Facilitates NilC Surface Exposure During Heterologous Expression in *Escherichia coli*

**FIGURE 1:**
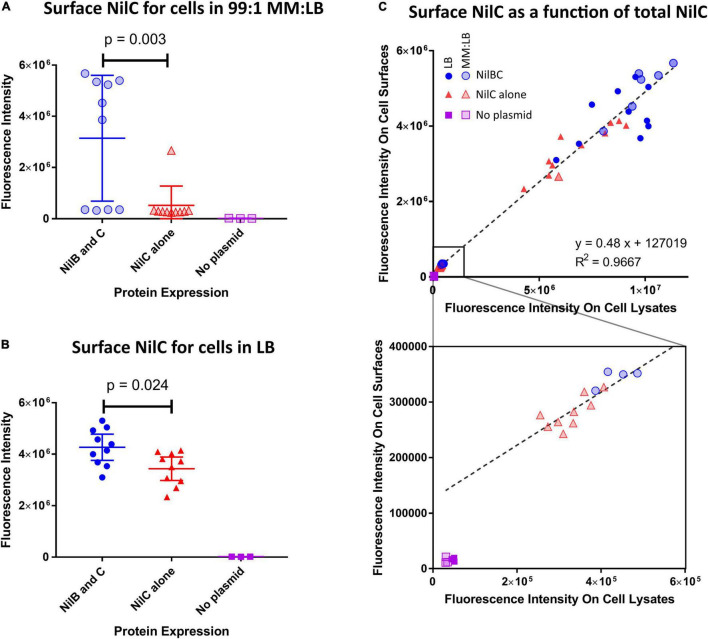
NilB positively influences the amount of NilC present on the surface of *E. coli* when co-expressed. *E. coli* BL21 DE3 C43 was transformed with pET-Duet-1 plasmids carrying *nilC* alone (red triangles) or both *nilC* and *nilB* (blue circles) under control of the inducible T7 polymerase promoter. A no-plasmid control (purple squares) was included to account for a potential cross-reacting signal. Surface exposure of NilC was quantified by immunofluorescence with anti-NilC antibody dot blotting of cells grown in LB [**(B,C)** solid symbols], or MM:LB [**(A,C)** transparent symbols]. *p*-values were determined *via* one-way ANOVA with Tukey’s multiple comparison. Total cellular levels of NilC were quantified by immunoblotting whole cell lysates from the same samples and surface NilC as a function of total cellular NilC is shown in **(C).** A linear regression of data from both media conditions revealed that total NilC expression was a strong predictor of surface exposure of NilC (R-squared = 0.9667). The bottom graph provides an expanded view of the region of the top graph indicated with a square.

To determine if NilC is a cargo protein for NilB, we monitored NilC surface exposure during heterologous expression in *Escherichia coli*, with and without co-expression with NilB. We constructed plasmids in which expression of *nilC* and, when present, *nilB* are under control of an IPTG-inducible T7 RNA polymerase (see section “Materials and Methods”). NilC surface exposure experiments were conducted by immuno-dot blotting with an anti-NilC antibody. As expected, *E. coli* without an expression plasmid did not react with the antibody. For NilC-expressing strains, we found that NilC was present on the surface of *E. coli* at significantly higher levels in the presence of NilB than in its absence after cultivation in either LB (Figure 1B and Supplementary Figure 1) or a minimal glucose medium supplemented with 1% LB (MM:LB) (Figure 1A). When considering all treatments together, we noted a positive correlation between the total amount of NilC expressed and the amount on the cell surface. This indicates that NilC can efficiently reach the *E. coli* cell surface even in the absence of NilB (Figure 1C). However, when NilC was expressed alone, its levels on the cell surface were 16.5% or 80% of those observed when co-expressed with NilB, during incubation in MM:LB or LB, respectively, indicating that NilB enhances the surface exposure of NilC. Furthermore, supernatants of induced *E. coli* cells expressing *nilC* with or without *nilB* did not have NilC levels detectable by the immuno-dot-blotting assay, indicating that cell lysis does not explain the surface NilC levels detected (Supplementary Figure 2). These data indicate that the presence of NilB supports greater overall levels of NilC expression in *E. coli* and that, like its TXISS_OMP_ family relatives, NilB facilitates surface exposure of its cargo: NilC lipoprotein. NilC surface exposure enhancement by, rather than complete dependence on, NilB TXISS_OMP_ is similar to the relationship between *N. meningitidis* fHbp and its TXISS_OMP_, Slam1. In either *E. coli* or *N. meningitides*, the fHbp lipoprotein can be surface exposed even in the absence of Slam1, particularly when expressed at high levels, but fHbp surface levels are elevated in the presence of Slam1 (Hooda et al., 2016; Fantappie et al., 2017; da Silva et al., 2019).

### NilC Is a Two-Domain Protein With a Predicted C-Terminal, 8-Stranded β-Barrel

**FIGURE 2:**
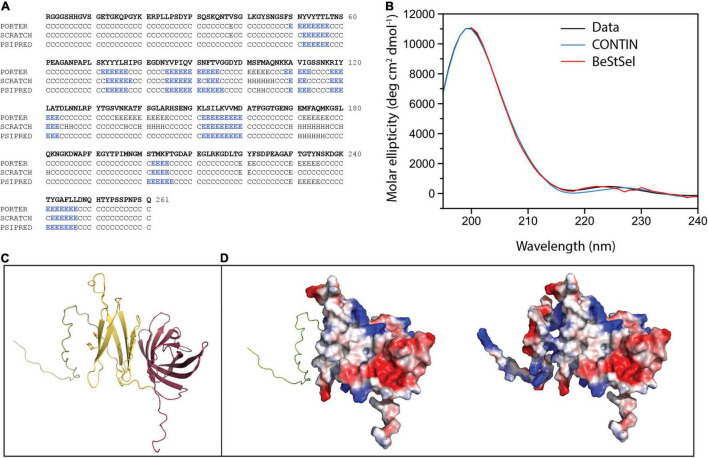
NilC secondary structure and structural models. **(A)** Secondary structure prediction for NilC_22–282_ was performed using Porter, Scratch, and Psipred algorithms (E β-strand, H α-helix, C random coil) with blue highlighting indicating the β-strand elements predicted by each. **(B)** A NilC_22–282_ CD spectrum was collected at 25°C in an AVIV model 420 CD spectrometer. Deconvolution of the spectra using two algorithms leads to excellent agreement with experimental measurement. **(C)** The NilC structural model showing disordered N-term (green), a relatively flexible N-terminal effector domain (yellow), and an 8-stranded barrel TXISS-targeting domain (purple). The two tyrosines conserved among *Xenorhabdus* NilC homologs are shown. **(D)** On the left, the electrostatic surface of effector and barrel domains (red, negative; blue, positive; and white, neutral charge densities) is shown, and, on the right, the electrostatic surface of entire protein, suggesting the charged N-terminal extension may help solubilize the protein despite the hydrophobic patch.

Given these data that indicate NilC is a surface exposed cargo protein for the NilB TXISS_OMP_, we sought to understand its molecular properties and biological function. Our investigation of the structural and biophysical characteristics of NilC required purified protein. A recombinant NilC soluble domain (NilC_22–282_) with a C-terminal 6X-His tag was expressed in *E. coli* and purified by nickel affinity and size exclusion chromatographies. The C-terminal His-tag did not impede the ability of NilC to function in colonization when it was expressed instead of the native sequence in *X. nematophila* (Supplementary Figure 3). The purified protein is remarkably highly soluble, remaining in solution until at least 80 mg/ml. Secondary structure prediction based on the NilC amino acid sequence indicates a protein that is largely random coil (∼71–74%) with only ∼24% of the protein predicted to form β-strand and little to no α-helix predicted (∼0–4%) (Figure 2A). This prediction was corroborated by circular dichroism (CD). The NilC_22–282_ CD spectrum is noteworthy for the fact that it has apparently no characteristic negative signal of α-helix (208 and 222 nm) or β-strand (218 nm) secondary structure elements (Figure 2B). On the other hand, the NilC spectrum has a positive band centered at 200 nm, which indicates it is not completely unfolded, as disordered proteins have a negative band at 195 nm. We suspected that a negative signal at 218 nm for β-strand might be masked in the experimental spectra by the high concentration of random coil with a positive signal at a nearby wavelength. CD spectrum deconvolution using CONTIN and BeStSel algorithms supported this interpretation and indicates a β-strand content between 43 and 49%, with no significant presence of α-helix (∼2%).

Tertiary structure predictions of NilC – both *via* well-established remote homology modeling in Phyre2 (Kelley et al., 2015) or *via* neural net analysis as implemented in RoseTTAFold (Baek et al., 2021), AlphaFold2 (Jumper et al., 2021), and their combined implementation in ColabFold (Mirdita et al., 2021) – predict with high statistical significance the 8-stranded C-terminal β barrel of the TX1SS targeting domain. The structure of this domain is robustly modeled using *Haemophilus haemolyticus* hemophilin (PDB: 6OM5) as a template (Latham et al., 2020). The N-terminal effector domain, on the other hand, is poorly defined even by these recent powerful structure prediction methods, although all models of the N-terminal domain have a large fraction of random coil, ∼7 β-strands or extended segments without well-defined secondary structure, and, essentially, no α-helical content (Figure 2C). These predictions are an excellent match to our experimental measurements. Intriguingly, a surface electrostatic calculation of the model with the highest amount of secondary structure shows a large non-polar patch, an unexpected feature for a protein so readily soluble (Figure 2D). Indeed, because the first 40 amino acids of the mature NilC sequence contain a high fraction of glycine and proline, we surmised they might be highly disordered. We thus created a second NilC construct, and expressed and purified NilC_62–282_. This protein is significantly less soluble than NilC_22–282_, bolstering the hypothesis that this N-terminal extension may protect the hydrophobic patch of purified NilC_22–282_ in solution. We also noticed an unusually high concentration of tyrosine in the primary sequence, with 12 Tyr in the first 133 amino acids of the secreted protein (9%) (Supplementary Figure 4C). Tyrosine is an important element in sugar-binding sites for its aromatic stacking and, to a lesser extent, -OH hydrogen bond-donating abilities (Quiocho, 1986; Weis and Drickamer, 1996; Hudson et al., 2015; Banno et al., 2017). The only homologs of *X. nematophila nilC* identified to date occur in two other species (of ∼23 with sequenced genomes) of nematode-associated *Xenorhabdus: X. innexi* and *X. stockiae*, both of which also contain the associated *nilB* gene (Grossman et al., 2021) (Supplementary Figures 4A,B). In each case, the NilC polypeptide is predicted to be a two-domain protein with a C-terminal barrel and conserved tyrosines at positions 75 and 77 of the mature sequence in the N-terminal presumed effector domain (Supplementary Figure 4C). Both of these tyrosines occur within a region predicted to be a β-strand (Figures 2A,C).

To further our experimental characterization of NilC_22–282_, we collected a 2D ^1^H-^15^N HSQC spectrum of ^15^N labeled protein. The nuclear magnetic resonance (NMR) spectrum shows good signal dispersion, covering a range of more than 4 ppm on the proton axis, indicating NilC is well-folded under the conditions used (Felli and Pierattelli, 2015; Supplementary Figure 5). The most intense peaks, which are collapsed into the center of the spectrum, imply that some parts of the protein are highly flexible. Finally, we carried out limited protease digestion of NilC_22–282_ in order to experimentally investigate the potential for stable subdomains. Several fragments of sizes between 12 and 27 kDa are relatively stable intermediate breakdown products of NilC (Supplementary Figure 6A). Mass spectrometry of the peptide mixture after partial digestion identified the prominent fragment as having a mass of approximately 12,358 Da (Supplementary Figure 6B). Mapping of this mass onto the possible array of all Proteinase K partial digestion fragments provides insight into the folded core of NilC. This mass corresponds to the C-terminal barrel without its last strand, implying once again that the two domains of NilC are structurally distinct, the first more open and flexible, the second stably folded (Figure 2C).

Taken together, these data present a consistent picture of non-acylated NilC as a highly soluble and folded two-domain protein formed of sections of β-structure and a large fraction of random coil. The C-terminal domain is an 8-stranded barrel, adding support to the hypothesis that this structural motif is the common feature of TXISS cargo proteins. On the other hand, all of our experimental results indicate the N-terminal domain does not adopt a very well-defined structure, and that, under the conditions tested and in the absence of any potential ligands, NilC includes flexible regions and protease-accessible sites.

### NilC Is Surface Exposed in *Xenorhabdus nematophila* Cells When Expressed at High Levels

**FIGURE 3:**
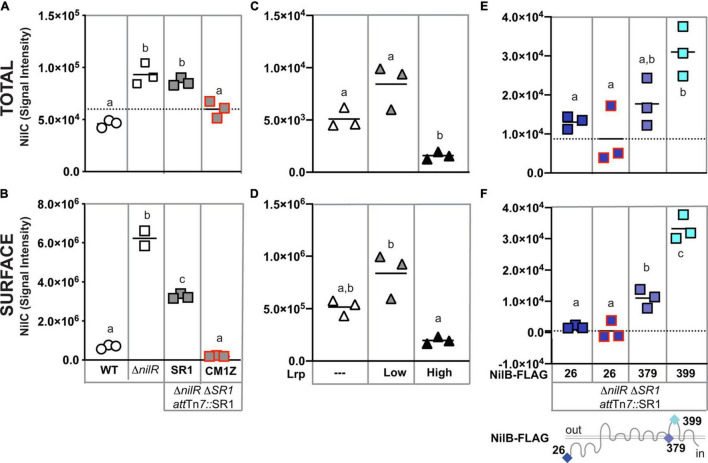
*Xenorhabdus nematophila* NilC is surface exposed by NilB when its expression is de-repressed. Cell lysate **(A,C,E)** or whole cell **(B,D,F)** preparations of LB-grown *X. nematophila* wild type (WT, circles), Δ*nilR* (squares), or Δ*lrp* (triangles) strains. At the *att*Tn*7* site of Δ*nilR*Δ*SR1* strains, wild-type SR1 (gray squares with a black outline) or SR1 with a *nilCM1Z* mutation (a red outline) and/or *nilB*-FLAG insertions (blue-shaded squares, sites of insertion noted beneath and on the NilB schematic bottom) were introduced **(A,B,E,F)**. Δ*lrp* strains were transformed with a vector-only control (white triangles) or plasmids expressing low or high levels of Lrp (gray and black triangles, respectively) **(C,D)**. Treatments were spotted onto nitrocellulose membranes. NilC was detected by immunoblotting with anti-NilC antibodies. Significantly different groups within each panel are indicated with different letters (tested using one-way ANOVA with Tukey’s *post hoc* multiple comparisons analysis). Dashed axis lines indicate the mean of the *nilCM1Z* datapoints **(A,E,F)**.

Given the evidence that NilC can be surface exposed in *E. coli* and that its C-terminal domain is predicted to form an 8-stranded barrel TXISS-targeting domain, we revisited the question of whether NilC is also surface exposed in the native context of an *X. nematophila* cell. Previous whole-cell protease-digestion data demonstrated periplasmic orientation of the lipoprotein NilC, based on the observation of protease resistance of NilC in the whole cell but not lysate samples of wild type *X. nematophila* (Cowles and Goodrich-Blair, 2004). Later, another protease digestion experiment to detect surface NilC was performed on an *X. nematophila*Δ*nilR* mutant, in which the absence of the transcription factor NilR causes *nilB* and *nilC* expression to be de-repressed (Cowles and Goodrich-Blair, 2006; Bhasin et al., 2012). In this analysis, slight shaving of NilC was detected in whole cells, indicating some surface exposure (Bhasin et al., 2012). To further examine *X. nematophila* NilC cellular localization, we used the same immuno-dot-blotting approach we had used for *E. coli* (Figures 3A,B). We assessed NilC levels in *X. nematophila* wild type compared to an isogenic Δ*nilR* mutant. In addition, we included an isogenic pair of Δ*nilR* strains in which the SR1 locus has been deleted from its native locus, and reintroduced, either as a wild type sequence, or including a *nilCM1Z* start-to-stop codon mutation, at the *att*Tn*7* site downstream of the conserved gene *glmS*, which is involved in peptidoglycan biosynthesis (Craig, 1991; Peters and Craig, 2001; Choi et al., 2005). The latter strain served as a negative control for NilC detection by an antibody.

In cell lysates (indicative of overall expression), we detected antibody reactivity in all strains tested, including the *nilCM1Z* negative control, indicating some cross-reactivity by the polyclonal antibody (Figure 3A). However, NilC-expressing strains had significantly higher antibody reactivity for whole cells than the *nilCM1Z* control, demonstrating the effective detection of NilC surface exposure using this method. When assessing levels of surface NilC in whole cell preparations of *X. nematophila*, we observed that wild type (*nilR*+) cells had little detectable NilC, while the Δ*nilR* strain had significantly higher signal intensity, indicative of surface NilC being present when *nilC* is de-repressed by deletion of *nilR*. Curiously, in strains in which SR1 had been introduced into the *att*Tn*7* site downstream of *glmS*, we observed a significant reduction in the amount of surface-exposed NilC detected relative to the isogenic parent Δ*nilR* strain, although total NilC levels were not significantly different (Figure 3B).

NilR synergistically represses *nilB* and *nilC* with another transcription factor, Lrp (Cowles and Goodrich-Blair, 2004, 2006). *X. nematophila* Lrp controls both mutualistic (with nematodes) and pathogenic (with insects) phenotypes. *X. nematophila* cells with fixed high levels of Lrp display higher levels of biofilm formation, nematode reproduction, and intestinal colonization, while *X. nematophila* cells with fixed low levels of Lrp display greater virulence in insects, compared to each other (Cowles and Goodrich-Blair, 2006; Hussa et al., 2015; Cao et al., 2017; Cao and Goodrich-Blair, 2020). We tested the impact of fixed high or low Lrp expression on NilC surface exposure and found that, as expected, NilC levels and surface exposure were inversely correlated with Lrp levels (Figures 3C,D); *X. nematophila* cells expressing high levels of Lrp had significantly lower levels of total and surface-exposed NilC relative to cells lacking or expressing low levels of Lrp.

Having demonstrated above that NilB facilitates NilC surface exposure during heterologous expression in *E. coli*, we endeavored to determine if this is also the case in the native *X. nematophila* context. We used immuno-dot blotting to examine NilC surface exposure in cells expressing select NilB FLAG-tag variants encoded by the SR1 locus integrated at the *att*Tn*7* site in a Δ*nilR*Δ*SR1* strain background (Figures 3E,F and Supplementary Figure 7). Our previous work using these and other FLAG-tag insertions across the length of the TXISS_OMP_ NilB revealed a topology consisting of a ∼138-amino acid N-terminal domain and 7 surface loops (Bhasin et al., 2012; Figure 3). A variant with a FLAG-tag insertion at the mature, periplasmically oriented N-terminus of NilB (FLAG-26), expresses detectable levels of NilB and functions as well as wild type in colonization. Another variant, with an insertion in a transmembrane helix (FLAG-379), does not express detectable levels of NilB and is insufficient for colonization (Bhasin et al., 2012). These two variants were used to compare surface levels of NilC surface exposure in the relative presence (FLAG-26) or absence (FLAG-379) of detectable NilB. Again, as was noted in other experiments, the total levels of NilC detected within cells correlated with the amount of NilC on the cell surface (compare Figures 3E,F). Surprisingly, significantly less surface NilC was detected in the FLAG-26 strain (that expresses detectable levels of NilB) relative to the FLAG-379 strain (which does not express detectable levels of NilB) during growth in LB. This indicates that a basal level of NilC can be surface exposed in the absence of NilB (Figure 3F), and that NilB can limit surface exposure of NilC. We next assessed the impact on NilC surface exposure of a FLAG-399 insertion in surface loop 6 of NilB. This variant expressed detectable NilB but is not functional in colonization. The FLAG-399 variant had significantly more surface NilC than either of the other two FLAG variants, suggesting that mutating the sixth extracellular NilB loop results in increased secretion of NilC cargo protein. A similar trend was noted when these same strains were grown in a minimal medium supplemented with casamino acids (Supplementary Figures 8A,B), although, in this case, the FLAG-26 expressing strain displayed relatively higher and more variable surface levels of NilC. As an alternate method of detecting surface NilC, *X. nematophila* whole cells grown in defined media supplemented with casamino acids and incubated with anti-NilC antibody (Cowles and Goodrich-Blair, 2004) and a fluorescent secondary antibody was observed using flow cytometry (Supplementary Figure 8C). Overall, these data using different growth conditions and immunodetection methods support the conclusion that, in *X. nematophila*, TXISS_OMP_ NilB can either inhibit or promote NilC surface presentation, relative to cells without NilB, which may suggest that TXISS_OMP_ activity is modulated depending on environmental conditions.

Taken together, the data described above are consistent with the model that NilB facilitates surface exposure of NilC, and that, when *nilB* and *nilC* expression is de-repressed by deletion of the transcription factors NilR or Lrp, NilC is surface exposed in *X. nematophila.* Our biophysical data indicate that, like other TXISS cargo proteins, NilC is a two-domain protein, with a C-terminal barrel domain proposed to be the TXISS targeting motif, and an N-terminal domain proposed to be a host-interaction effector. To further explore the possible effector role of *X. nematophila* NilC, we took a two-pronged approach. In the first approach, we used histochemistry to examine the molecules that are specifically presented on the host nematode intestinal surface, where *X. nematophila* adheres in a manner positively influenced by NilB and NilC (Chaston et al., 2013). In the second, we compared the proteomes and metabolomes of *X. nematophila* strains with and without SR1 to identify metabolic pathways and activities that may be impacted by the presence or absence of NilB and NilC.

### The *Steinernema carpocapsae* Anterior Intestinal Cecum Expresses a Wheat-Germ Agglutinin-Reactive Material That Contributes to SR1-Mediated *Xenorhabdus nematophila* Colonization

**FIGURE 4:**
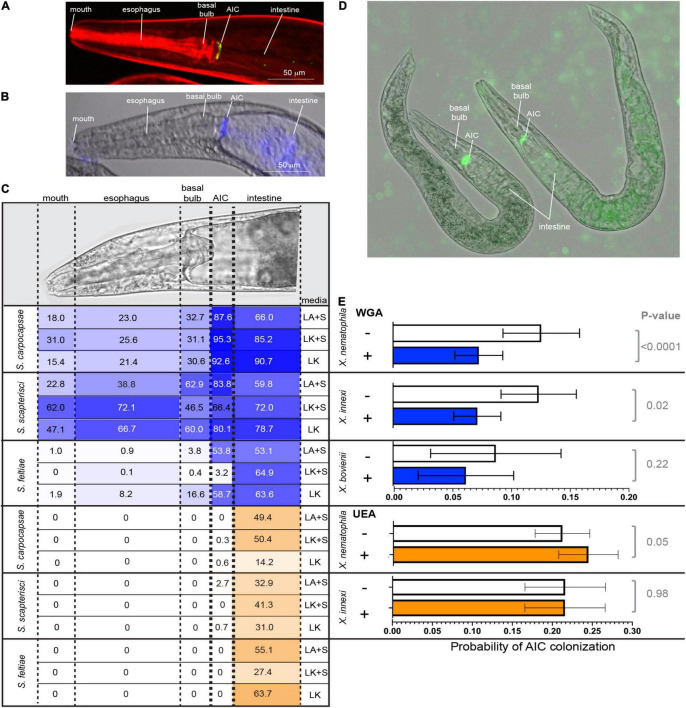
**(A)** *S. carpocapsae* nematode colonized at the anterior intestinal cecum (AIC) by *X. nematophila* expressing green fluorescent protein. Nematode tissue is stained with rhodamine phalloidin. **(B)** False color overlay (a bright field and fluorescence) of uncolonized *S. carpocapsae* grown on liver kidney agar without symbiont, and then incubated with F-WGA (a blue color) for 24 h before imaging. **(C)** At the top is shown a DIC image of *S. carpocapsae* with assayed body parts (mouth, esophagus, basal bulb, AIC, and intestine), roughly defined by dashed lines. *S. carpocapsae, S. scaptersici*, and *S. feltiae* were grown on lipid agar or liver kidney agar (LA or LK) with their respective symbionts (+S) or on liver kidney agar without their symbionts. Nematodes were incubated for 24 h with either F-WGA (blue, top section) or F-UEA (orange, bottom section) before observation for lectin binding to individual body parts. The percentage of all observed nematodes that had lectin binding at that body part is indicated within each cell, and is also represented by the intensity of shading (darker shading = higher percentage). WGA, but not UEA, reproducibly binds to the AIC, with the highest frequencies observed in *S. carpocapsae* and *S. scapterisci* grown on lipid agar with their respective symbionts. **(D,E)** Competition experiments were conducted in which nematodes cultivated on liver kidney agar without their symbionts were exposed to WGA, UEA, or a PBS control and with their green fluorescent protein-expressing *Xenorhabdus* symbiont. After 24 h, individual nematodes were imaged and assessed for colonization at the AIC. **(D)** Shows *X. nematophila* AIC colonization within two *S. carpocapsae* nematodes incubated without lectin (PBS control). **(E)** Probability of AIC colonization by the symbiont of each nematode (*X. nematophila* colonizing *S. carpocapsae, X. innexi* colonizing *S. scapterisci*, and *X. bovienii* colonizing *S. feltiae*) after incubation without (–; white bars) or with (+) WGA (blue bars) or UEA (orange bars). The probability of AIC colonization in *S. carpocapsae* and *S. scapterisci*, but not *S. feltiae*, was significantly affected by treatment with WGA (*S. carpocapsae*: *F*_3,59_ = 3.91, *p* = 0.01; *S. scapterisci*: *F*_1,72_ = 5.37, *p* = 0.02; *S. feltiae*: *F*_1,32_ = 1.5, *p* = 0.23), but not UEA (*S. carpocapsae*: *F*_1, 2684_ = 3.70, *p* = 0.05; *S. scapterisci*: *F*_1,2696_ = 0, *p* = 0.9833).

*Xenorhabdus* bacteria colonize the anterior intestinal cecum (AIC) of their *Steinernema* nematode hosts during the reproductive juvenile and adult stages (Figures 4A,D). The AIC is immediately posterior to the basal bulb, a pumping organ that drives ingestion from the mouth, through the esophagus, and into the intestine (Figure 4A). To begin investigating the surface chemistry of the AIC bacterial colonization site, we selected three nematodes: *S. carpocapsae*, *S. scapterisci*, and *S. feltiae*. The symbionts of the first two nematodes, *X. nematophila* and *X. innexi*, respectively, both encode NilB and NilC homologs (Supplementary Figure 4). The symbiont of *S. feltiae, X. bovienii*, does not. Adult nematodes that had been raised in the presence or absence of their bacterial symbiont were treated with lectin fluorescent conjugates and observed by fluorescence microscopy for lectin binding to host tissues, monitoring frequency of binding to the mouth, the esophagus, the basal bulb, the AIC, and the intestine (Figure 4B). We found that all lectins tested had some binding to various tissues in all three nematodes tested. Binding varied according to the media type (lipid agar or liver kidney agar) as well as the presence/absence of bacterial symbiont (Supplementary Data File 1 and Supplementary Figure 9).

We focused our attention on fluorescent conjugate wheat germ agglutinin (F-WGA, which reacts with *N*-acetyl glucosamine or *N*-acetyl neuraminic acid), because this lectin had consistent reactivity with *S. carpocapsae* nematode tissues, and because it previously had been observed to react with material extruded from the intestinal receptacle colonization site of the infective juvenile stage *S. carpocapsae* nematode (Martens et al., 2005). As a control, we included fluorescent conjugate Ulex Europaeus Agglutinin (F-UEA), which reacts with alpha-linked fucose. Observed individuals from all three nematode species from all three cultivation conditions displayed both F-WGA and F-UEA localization to the intestine (Figure 4C). In contrast, F-WGA but not F-UEA localized to the AIC. F-WGA localization to the AIC occurred in *S. carpocapsae* and *S. scapterisci*, but not *S. feltiae* consistently at high frequency (88–95%, 66–84%, and 3–59%, respectively), indicating that the two nematode hosts of SR1-encoding symbionts consistently express F-WGA-reactive material at the symbiont colonization site, while the host of the non-SR1-encoding symbiont does not (Figure 4C).

Given that both F-WGA and the respective *Xenorhabdus* bacterial symbionts are able to bind to the AIC of both *S. carpocapsae* and *S. scapterisci* in the majority of the nematode population, we hypothesized that the WGA-reactive material might be important for colonization of their bacterial symbionts as a binding ligand, a nutrient source, or both. We reasoned that if the WGA-reactive material is involved in colonization, that the addition of soluble WGA to adult nematodes would block bacterial interaction with the WGA-reactive material on the AIC surface. To test this idea, we exposed adult nematodes simultaneously to unconjugated WGA and GFP-expressing bacterial symbionts for 24 h before monitoring by fluorescence microscopy the presence of bacterial colonization at the AIC (Figure 4D). The presence of WGA significantly reduced the presence at the AIC of GFP-expressing *Xenorhabdus* symbionts in both *S. carpocapsae* (*p* < 0.0001; note that composite data including both wild type and Δ*SR1* treatments were included in this analysis) and *S. scapterisci* (*p* = *0.02*) but not in *S. feltiae* (*p* = *0.23*) when compared to a control that was exposed only to the GFP-expressing bacterial symbionts (Figure 4E). In contrast, the presence of UEA did not decrease the probability for either *X. nematophila* or *X. innexi* to colonize their nematode hosts (*S. feltiae* was not examined) (Figure 4E).

We next considered the possibility that the Nil proteins are responsible for interaction with the AIC WGA-reactive material. If so, then we predict that any Nil-independent adherence to the AIC would not be inhibited by WGA. To test this idea, we analyzed the data to discern the influence of the presence or absence of the SR1 locus on *X. nematophila* colonization of *S. carpocapsae* nematodes exposed or not to WGA. The probability of *X. nematophila* colonization of the *S. carpocapsae* AIC was significantly higher (*p* < 0.05) for SR1 + treatments (0.114 ± 0.022 and 0.146 ± 0.027 with and without WGA, respectively), relative to Δ*SR1* treatments (0.095 ± 0.019 and 0.083 ± 0.017, with and without WGA, respectively). Also, consistent with the role for SR1 in mediating interactions with the WGA-reactive material, the probability of AIC colonization by Δ*SR1 X. nematophila* was not significantly altered by the presence of WGA (*p* = 0.36).

**FIGURE 5:**
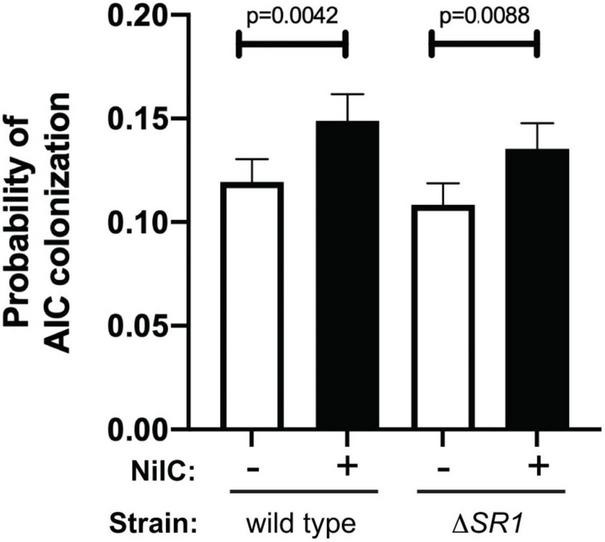
Addition of soluble, purified NilC increases colonization of the anterior intestinal cecum. Mean with standard error of the probability of AIC colonization is shown. Green-fluorescent protein expressing *X. nematophila* wild type or Δ*SR1* mutant was exposed for ~24 h to *S. carpocapsae* nematodes in a buffer with (+) or without (–) purified, soluble NilC protein. Nematodes were observed by microscopy for the presence or absence of *X. nematophila* at the anterior intestinal cecum (AIC). A total of 8,384 observations were made and analyzed using PROC GLIMMIX of SAS to determine if treatment combination (NilC and strain) differed in probability for colonization. There were differences in probability for colonization (*p* = 0.0004), and there was significantly higher AIC colonization in the presence of NilC for both wild type and Δ*SR1*.

We hypothesized that *X. nematophila* surface-exposed NilC interacts with molecules on the AIC surface. If so, then we predicted that, as with WGA, the addition of free, soluble NilC would inhibit bacterial interaction with the AIC. We exposed nematodes (cultivated in the absence of their symbiont) to purified NilC protein and GFP-expressing *X. nematophila* cells with or without the SR1 locus. In this experiment, the isogenic Δ*SR1 X. nematophila* strains with and without the SR1 locus at the *att*Tn*7* site colonized to similar levels, indicating that the conditions of the experiment were insufficient to distinguish the two strains. However, for both strains, we observed that nematodes exposed to NilC exhibited significantly (*S. carpocapsae* WT *p* = 0.0042, Δ*SR1 p* = 0.0088) higher rates of colonization compared to nematodes exposed to just the GFP-expressing symbionts (Figure 5). This finding was contrary to our prediction and may indicate that free NilC can facilitate colonization, perhaps by signaling for, or directly catalyzing, release of a nutrient from the host-cell surface. To pursue this hypothesis, we created an *X. nematophila* strain, expressing a non-lipidated version of NilC (generated by the introduction of two lipobox mutations: V17A and C20S). This strain was unable to colonize the infective juvenile stage of *S. carpocapsae* nematodes (Supplementary Figure 3). Indeed, unlike wild type NilC, the V17A-C20S NilC protein was not detected on the *X. nematophila* cell surface, and was only detected in the supernatant in 2/6 replicate samples (Supplementary Figure 10). These data suggest that non-lipidated NilC is predominantly retained within the periplasm.

### Deletion of *nilB* and *nilC* Causes Global Metabolome and Proteome Changes Including Impacts on a Peptidoglycan Precursor and Exopolysaccharide Biosynthesis

Based on the data described above, our working model is that NilB is a TXISS_OMP_ that conditionally facilitates surface exposure of the lipoprotein NilC, which is a host-interaction effector. Since external treatment with WGA inhibits *X. nematophila* interaction at the nematode AIC surface, while soluble NilC enhances this interaction, we infer that NilC either interferes with a host defense pathway, as for the TXISS cargo factor H-binding protein, or helps to acquire a host-derived nutrient, similar to the function of TXISS lipoprotein effectors transferrin and lactoferrin-binding proteins. To gain insights into downstream physiological effects of NilC action, we compared the proteomes and metabolomes of *X. nematophila*Δ*nilR*Δ*SR1* emptyTn*7* relative to the Δ*nilR*Δ*SR1* Tn*7*:*SR1* (for this comparison, referred to as Δ*SR1* and WT, respectively) (Supplementary Table 1). We grew the *X. nematophila* cells in a minimal medium with glucose as a carbon source (Supplementary Figure 11) and harvested cells and supernatant at OD_600_ ∼0.6 for processing and analysis. Whole cell and supernatant samples were analyzed by liquid chromatography tandem mass spectrometry (LC-MS/MS) for proteomic analysis and ultra-high-performance liquid chromatography high-resolution mass spectrometry (UPLC-HRMS) for metabolomic analysis.

**FIGURE 6:**
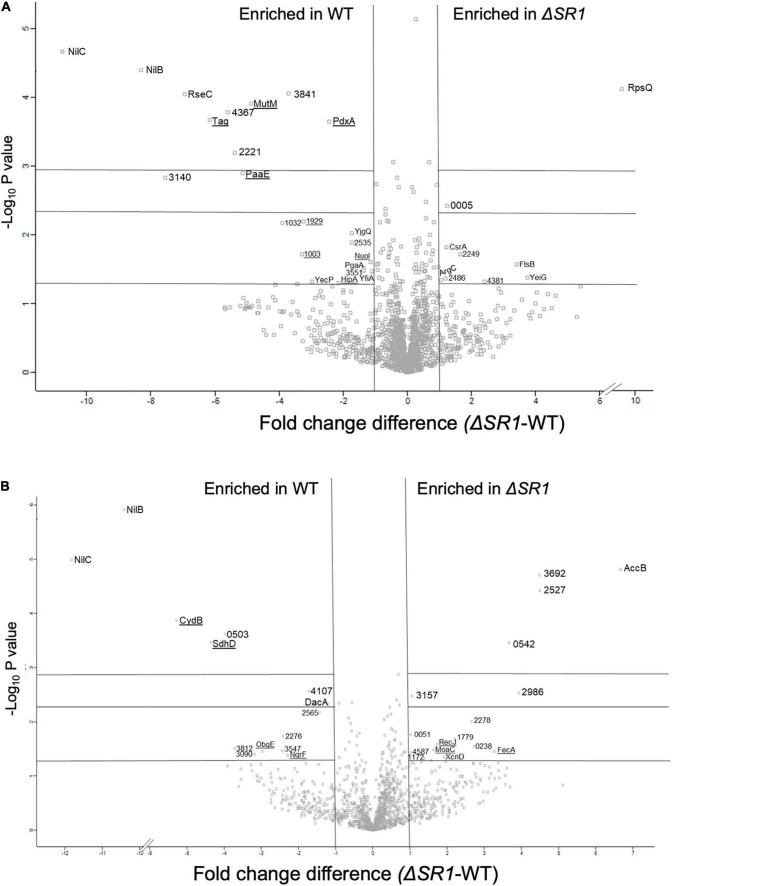
Differentially translated proteins between Δ*SR1* and WT show significant differences in metal-related activities (binding, homeostasis, and metabolic pathways). Volcano plot representations of proteins that are differentially present in *X. nematophila* wild type and Δ*SR1* strains for **(A)** whole cell fraction and **(B)** supernatant fraction. Proteins indicated on the left of the vertical lines were detected at significantly higher levels (| FC| > 1) in wild type, while those listed to the right were detected at significantly higher levels (| FC| > 1) in the Δ*SR1* mutant. Underlined proteins represent proteins with expected metal-binding activity. Significance values (from bottom to top) are indicated by the horizontal lines at *p* < 0.05, 0.005, and 0.001.

In the proteomics comparison of WT and Δ*SR1*, 3,336 proteins were detected in total: 1,742 proteins in whole cell samples and 1,594 proteins in the supernatant samples. Of the 3,336 proteins detected, 61 were considered to be significantly different in abundance between Δ*SR1* and WT based on a Student’s *T*-test filter (*p* < 0.05) and a fold change filter (|FC| > 1) (Supplementary Tables 2, 3 and Figures 6A,B). In the metabolomics dataset, 85 metabolites were identified in total, and comparisons were made between the metabolomes using partial least square discriminant analysis (PLS-DA) to determine the separation of the strain metabolic profiles (Supplementary Figure 12). For both the supernatant and whole cell fractions, there is clear separation of the metabolic profiles between Δ*SR1* and WT. Student’s *t*-tests were applied to determine significant metabolites between strains. For the whole cell fraction, 18 metabolites were significant (*p* < 0.1) and 10 metabolites were significant (*p* < 0.1) for the supernatant fraction (Supplementary Data File 2 and Supplementary Figure 12C).

**FIGURE 7:**
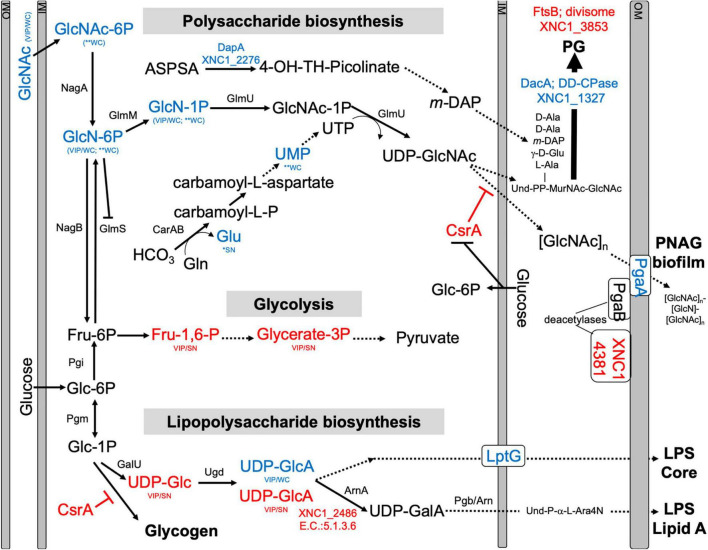
Predicted pathways for amino sugar metabolism leading to peptidoglycan (PG), exopolysaccharide [poly-*N*-acetylglucosamine (PNAG)], lipopolysaccharide LipidA biosynthesis, and glycolysis pathways. Blue and red font texts indicate higher and lower abundance of the indicated metabolites and proteins in WT relative to Δ*SR1*. Asterisks (*) indicate significant differences based on Student’s *T*-test, while VIP indicates VIP > 1 in PLS-DA plots, with WC and SN indicating whether the observed difference was in a whole cell or supernatant, respectively. XNC1_4381 shows similarity to PgaB deacetylase, and XNC1_2486 is predicted to convert UDP-glucuronate (UDP-GlcA) to UDP-galacturonate (UDP-GalA), similar to the enzyme ArnA. GlcNAc-6P, *N*-Acetyl-Glucosamine; GlcN-6P, Glucosamine-6-phosphate; GlcN-1P, Glucosamine-1-phosphate; GlcNAc-1P, *N-*Acetyl-Glucosamine-1-phosphate; UDP-GlcNAc, Uridine diphosphate *N-*acetyl glucosamine; Und, Undecaprenyl; MurNac, *N-*Acetyl-Muramic Acid; Fru-6P, Fructose-6-phosphate; Fru-1,6-P, Fructose 1,6 bisphosphate; Glycerate-3P, 3-Phosphoglycerate; Glc-6-P, Glucose-6-phosphate; Glc-1P, Glucose-1-phosphate; UDP-Glc, Uridine diphosphate glucose; UDP-GlcA, Uridine diphosphate glucuronate; UDP-GalA, Uridine diphosphate galacturonate; L-Ara4N, *L*-4-aminoarabinose. Dashed lines indicate multiple steps in the indicated pathway.

Among the metabolites with elevated abundance in the WT strain were the amino sugars *N*-acetylglucosamine 1/6-phosphate, glucosamine phosphate, and the nucleotide sugar UDP-glucuronate, and, from the PLS-DA analysis (VIP > 1), the nucleotide amino sugar UDP-*N*-acetylglucosamine, the precursor to the exopolysaccharide poly-β - 1,6-*N*-acetylglucosamine (PNAG) (Figure 7 and Supplementary Figure 12). UDP-glucuronate is a central intermediate in the synthesis of precursors for exopolysaccharide and cell wall polysaccharide biosynthesis. The proteome revealed that a putative UDP-glucuronate epimerase (XNC1_2486) (Borg et al., 2021) has lower abundance in WT relative to Δ*SR1* (Supplementary Table 2), consistent with the elevated UDP-glucuronate abundances detected in the former. Amino sugars are involved in peptidoglycan and exopolysaccharide biosynthesis and can be used by bacteria as sources of carbon and nitrogen by catabolism through glycolysis (Dobrogosz, 1968). Consistent with this metabolic connection, according to the PLS-DA analysis, relative to Δ*SR1*, whole cell samples of WT had higher abundances of the glycolytic intermediates 3-phosphoglycerate and fructose 1,6-bisphosphate, as well as UDP-glucuronate and UDP-glucose, lipopolysaccharide precursors. The proteomic data also indicated differences between the WT and Δ*SR1* strains in polysaccharide and glycolytic processes.

The differentially abundant proteome included XNC1_2986, predicted to encode a sugar-phosphate-binding transcriptional regulator of the RpiR family, which was more abundant in the Δ*SR1* mutant relative to WT. In addition, LptG (XNC1_4255), a component of the ABC transporter that exports lipopolysaccharide across the inner membrane (Ruiz et al., 2008), was at higher abundance in wild type relative to Δ*SR1.* DacA, a predicted D-alanyl-D-alanine carboxypeptidase that removes the C-terminal D-alanyl from sugar-peptide cell wall precursors during peptidoglycan biosynthesis, was at higher abundance in the WT relative to Δ*SR1*. FtsB, a cell division protein that regulates peptidoglycan biosynthesis, was lower in WT relative to Δ*SR1* (Boes et al., 2019). WT had higher abundance of a PgaA (a.k.a. HmsH) homolog, the secretin for the exopolysaccharide PNAG (Drace and Darby, 2008; Low and Howell, 2018). The mutant had elevated abundance of CsrA, which regulates PNAG biofilm formation negatively and positively in *E. coli* and *Yersinia pestis*, respectively (Parker et al., 2017; Berndt et al., 2019; Silva-Rohwer et al., 2021), and XNC1_4381, predicted to encode a lipoprotein with poly-β-1,6-*N*-acetylglucosamine de-*N*-acetylase activity, similar to PgaB/HmsF (Calder et al., 2020). The *pga* locus is necessary for *X. nematophila* (and *Yersinia pestis*) biofilm formation on the external surfaces of *Caenorhabditis elegans* nematodes, but is not necessary for colonization of *S. carpocapsae* infective juvenile nematode receptacles (Couillault and Ewbank, 2002; Drace and Darby, 2008). Overall, the combined proteomics data indicate that the flux of amino sugar intermediates toward peptidoglycan, exopolysaccharide, and lipopolysaccharide structures is altered in the Δ*SR1* mutant relative to wild type (Figure 7).

Biofilm formation can be regulated by c-di-GMP, levels of which are affected by the pool of purine nucleotides (Kim et al., 2014). Among the metabolites that were differentially abundant between the WT and Δ*SR1* were those involved in purine metabolism. Xanthosine, AICAR, guanine, and guanosine were detected at significantly higher abundances in the WT background whole cell fraction than in the corresponding Δ*SR1* fraction (Supplementary Figure 12). Metal binding proteins involved in regulating DNA repair, transcription, and translation are differentially abundant between strains, coinciding with the significant purine and pyrimidine metabolites found in the metabolomics analysis. Tag (XNC1_4499), MutM (XNC1_0165), and RecJ (XNC1_1136) are proteins involved in the base-excision repair (BER) pathway. RecJ binds Mg, Mn, and Co and has a significantly higher abundance in the Δ*SR1* mutant in the supernatant fraction. The BER pathway repairs DNA damage caused by oxidation, alkylation, and deamination, and their differential abundance may indicate differences in DNA damage occurring in these strains (Krokan and Bjoras, 2013).

**FIGURE 8:**
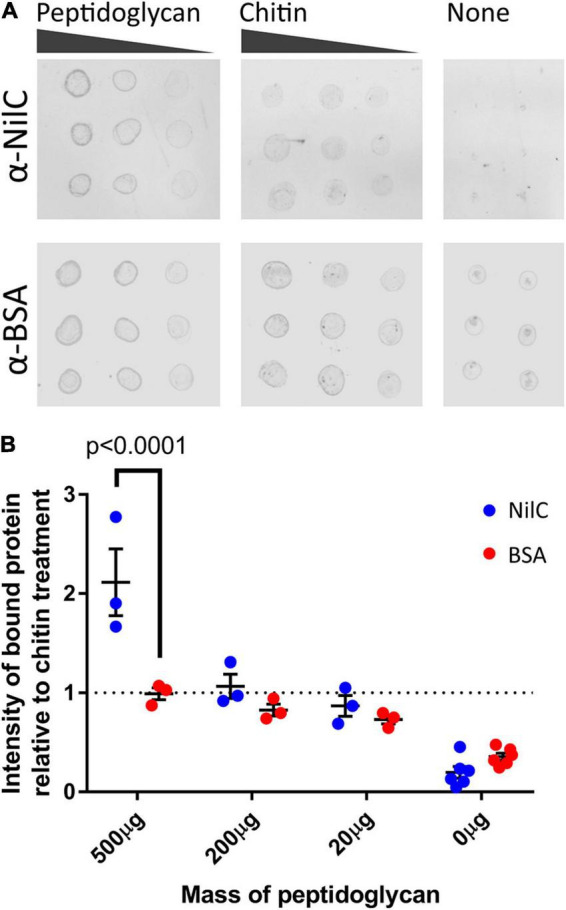
NilC binds peptidoglycan. **(A)** Immuno-dot blots depicting the amount of NilC or bovine serum albumin (BSA) bound to *B. subtilis* peptidoglycan or chitin after an hour of incubation. Three different concentrations of peptidoglycan and chitin were used to provide a gradient (horizontal dots), and 2 μl of each reaction were spotted in triplicate (vertical dots). Immunoblots were probed with the indicated primary antibodies and then labeled with an α-rabbit IgG antibody conjugated to a 680-nm fluorophore for imaging. **(B)** To visualize peptidoglycan binding, protein intensity values were normalized to the average intensity of protein in the chitin samples and plotted. Error bars depict the standard error of each mean. A Tukey’s honest significance test was used to compare NilC and BSA intensities; a Dunnett’s test was used to compare peptidoglycan-associated protein intensities to chitin-associated protein intensities.

The metabolomics and proteomics analysis described above suggests that the presence of NilB and NilC influences polysaccharide homeostasis. This, combined with the ability of NilC to increase *X. nematophila* colonization at a glycan-rich tissue and our observations of slight structural similarity between NilC and CBM domains (Supplementary Figure 4), led us to hypothesize that NilC might have binding and/or cleavage activity for polysaccharides. We tested this in preliminary assays by assessing the ability of purified NilC to bind to chitin-coated beads or to cleave various disaccharide substrates or polysaccharides using fluorogenic substrates with negative results in both cases. We next tested if NilC can bind peptidoglycan (PG), which it would encounter when periplasmically oriented. We chose to use commercially available PG purified from the Gram-positive bacterium *Bacillus subtilis* to avoid potential complications of contaminating lipopolysaccharide from Gram-negative bacterial sources. BSA or NilC was incubated with PG or chitin as a negative control, and the levels of protein that pelleted with these insoluble substrates were quantified (Figure 8). We found that, at the highest concentration of polysaccharide tested (500 mg/ml), NilC associated with PG significantly more than with chitin (*p* = 0.0454). Furthermore, NilC associated with PG significantly more than did BSA (*p* < 0.0001). These data suggest that, when in the periplasm, NilC may bind to peptidoglycan.

## Discussion

**FIGURE 9:**
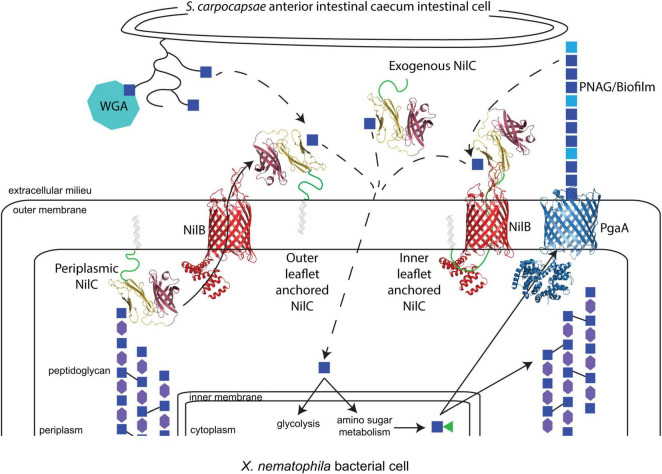
The model of potential molecular interactions occurring during *X. nematophila* colonization of *S. carpocapsae* anterior intestinal cecum epithelial cell surfaces. *N*-acetyl-glucosamine (GlcNAc; blue squares) is present on the *S. carpocapsae* epithelial cell surface, possibly as part of mucin proteins (curved lines), as demonstrated by the binding of wheat germ agglutinin (WGA; teal octagon) to this tissue. GlcNAc is also a component of bacterial peptidoglycan, alternating in β-1,4 linkage with *N*-acetylmuramic acid (MurNAc; purple hexagons) and crosslinked to other polysaccharide chains through peptide linkages (black solid lines). *X. nematophila* encodes homologs of the Pga synthase-dependent exopolysaccharide secretion system for the production of poly-β-1,6-*N*-acetyl-glucosamine (PNAG), composed of GlcNAc and de-acetylated GlcN (light blue squares). A working model based on evidence presented in this study is that periplasm-oriented NilC binds through its N-terminal effector domain (yellow) to GlcNAc residues within peptidoglycan, possibly modulating turnover. NilC can be extracellular, and its transport across the outer membrane by NilB (red) is directed by virtue of its C-terminal β-barrel TXISS-targeting domain (purple). The lipid anchor of NilC may be embedded either in the inner or outer leaflet of the outer membrane. In the former possibility, the long, disordered N-terminal region of NilC (a green cartoon line) may thread through the barrel of NilB, analogous to the structural interactions between the lipoprotein RcsF and associated OMPs (Rodriguez-Alonso et al., 2020). Extracellular NilC may bind to nematode or bacterial glycan-derived GlcNAc and facilitate its uptake (dashed arrows). NilB (red) and NilC models are predictions from Phyre2.0 and AlphaFold, with the N-terminal random coil of NilC represented as a green cartoon line. The structure of PgaA (blue) is PDB 4Y25 (Wang et al., 2016).

Recent discoveries have revealed that members of the DUF560 family of OMPs are Type XI secretion systems (TXISS) that can move proteins across the outer membrane (Hooda et al., 2016, 2017; Fantappie et al., 2017; da Silva et al., 2019; Bateman et al., 2021; Grossman et al., 2021). TXISS-dependent effectors include binding proteins for transferrin, lactoferrin, factor H, and heme. However, the full range, characteristics, and functions of TXISS cargo remain to be explored. Here, we demonstrate that the lipoprotein NilC, a mutualism species-specificity factor, is a cargo protein for the TXISS_OMP_, NilB (Heungens et al., 2002; Cowles and Goodrich-Blair, 2004, 2008; Bhasin et al., 2012; Chaston et al., 2013; Grossman et al., 2021). Our biophysical data confirm the emerging concept that the TXISS cargo is characterized by an N-terminal functional domain that mediates host interactions and a C-terminal domain that targets cargo proteins for secretion by TXISS. Hooda et al. (2017) noted divergent functional N-terminal domains, and a common C-terminal barrel domain of the known TXISS cargo proteins TbpB, HpuA, and fHbp for which structural data were available at that time (Hooda et al., 2017). We now add experimental and bioinformatics data that show this structural framework extends to a TXISS cargo protein with a distinctive N-terminal domain and is thus more generalizable than previously realized. We found that the N-terminal 40 amino acids of NilC are disordered, supporting the emerging concept that many lipoproteins destined for the outer membrane have a long, disordered linker at their N-terminus (El Rayes et al., 2021). In *E. coli*, this linker is important for trafficking by the Lol (localization of lipoproteins) system and for appropriate surface localization of the four extracellular lipoproteins tested, including RcsF (El Rayes et al., 2021). Recent structural data have indicated that RcsF associates with BamA before being transferred to a nascent OMP that is folded by the Bam complex. In this model, the RcsF lipid anchor is embedded in the inner leaflet of the outer membrane, with the long, disordered region threading through the OMP to the surface exposed remainder of the protein (Rodriguez-Alonso et al., 2020). A similar assembly process and resulting topography may also occur for NilC and TXISS (Figure 9).

According to the emerging two-domain model for the TXISS cargo, the NilC N-terminal protein sequence between the disordered linker and the C-terminal β-barrel likely comprises the functional effector domain. The molecular function of this effector presumably facilitates mutualistic colonization of the nematode host intestine, for which NilC is required. Some TXISS cargo proteins (e.g., TbpB) are co-receptors for nutrient uptake systems (Eisenbeis et al., 2008; Konopka, 2012; Pogoutse and Moraes, 2017), while another (fHbp) protects from host immunity (Schneider et al., 2006). Our data do not yet distinguish between these two possible functions for NilC. Indeed, our data raise a third possibility that the TXISS cargo can have both periplasmic and extracellular functions. Our demonstration that NilC can bind purified bacterial peptidoglycan suggests that periplasmic NilC binds to the cell wall (Figure 9), and is the first experimental indication that NilC may be a carbohydrate binding protein. In this context, we noted an intriguing abundance of tyrosine residues in the N-terminal effector domain. Carbohydrate-aromatic amino acid contacts are a defining characteristic of non-covalent protein carbohydrate-binding pockets due in particular to the favorable CH-π interactions, with tyrosine being the second most frequently carbohydrate-contacting aromatic amino acid (Quiocho, 1986; Weis and Drickamer, 1996; Hudson et al., 2015). A 62-residue stretch of NilC aligns (albeit with low confidence) with the carbohydrate-binding module (CBM) 4/9 domain d1guia (29% identity over 62 residues) (Supplementary Figure 4D). While different CBM4 domains recognize diverse glycans using tryptophan or tyrosine residues, in at least one example, the CH-π - binding pocket interactions are donated entirely by tyrosines (Boraston et al., 2002). Conserved tyrosine residues are involved in peptidoglycan peptide recognition in both bacterial lysostaphin (Mitkowski et al., 2019), and eukaryotic peptidoglycan recognition protein-Iα (Guan et al., 2004).

What role, if any, periplasmic NilC plays in nematode host colonization remains to be investigated. *X. nematophila* expressing a V17A-C20S NilC mutant variant is colonization deficient and was not detected consistently in the supernatant, suggesting that, without its covalently attached lipid, this variant NilC is retained in the periplasm. This may be due to sequestration by binding to peptidoglycan, or due to lower efficiency of secretion through TXISS. Regardless, these data argue against a function for periplasmic NilC in facilitating nematode colonization, but do not preclude the possibility that it plays some other cellular role. Periplasmic NilC may modulate peptidoglycan remodeling or stability, activities that could explain the influence of SR1 on polysaccharide and glycolytic metabolism revealed by this study. Similar effects have been observed for the major outer membrane lipoprotein, Lpp, which is predominantly located in the bacterial periplasm where it binds peptidoglycan, and is situationally surface exposed. (Narita and Tokuda, 2011). The predominant function of periplasmic Lpp is in cell envelope structure maintenance. The route by which Lpp is surface exposed and its function, if any, at that location are poorly understood, but are postulated to occur as part of a stress response (Konovalova and Silhavy, 2015; Bahadur et al., 2021; Winkle et al., 2021). Regardless, our findings here suggest that periplasm-surface duality of lipoproteins may be more common than currently appreciated.

The possibility that extracellular NilC serves as the nematode-host interaction effector is supported by our finding that the exogenous addition of soluble, purified NilC can increase *X. nematophila* colonization of the nematode anterior intestinal cecum. If extracellular NilC binds carbohydrates, as suggested above, its substrate may be derived from this surface, which we show here is coated in glycans, including either GlcNAc (a component of peptidoglycan) or *N*-acetylneuraminic acid (Guerardel et al., 2001). Although the glycomes of *Steinernema* nematodes have yet to be elucidated, other nematodes lack *N*-acetylneuraminic (sialic) acid (Bacic et al., 1990), and the intestinal mucus layer is composed, in part, of O-linked serine/threonine glycosylated proteins (mucins) with terminal GlcNAc residues. This mucus layer is part of the nematode host-pathogen interface, and is considered part of the immune defense system. In *C. elegans*, mucins are upregulated in response to *Pseudomonas aeruginosa* infection and elicit downregulation of *P. aeruginosa* metal-binding siderophores and biofilm formation (Troemel et al., 2006; Head et al., 2017). In turn, *P. aeruginosa* can counteract these effects and exploit mucins on *C. elegans*’ intestinal surfaces to increase virulence (Wheeler et al., 2019; Hoffman et al., 2020). Specifically, *P. aeruginosa* colonization and biofilm formation are increased by the presence of *N-*acetyl-galactosamine monosaccharides derived from the Mul-1 mucin (Hoffman et al., 2020). Similarly, *X. nematophila* NilC may be involved in sensing host-derived monosaccharides to promote colonization and biofilm formation, although, in this case, to facilitate a mutualistic association.

The interface between a host and bacterium is not exclusively composed of host-derived glycans, and extracellular NilC may promote host interactions through binding of a bacterial-derived carbohydrate, such as an exopolysaccharide component of a surface-adherent biofilm. We identified several links between NilB and NilC and biofilm exopolysaccharides. Our analysis of the global transcription factor Lrp, the regulon of which includes *nilC*, revealed an inverse relationship between NilC surface levels and glass surface biofilm formation, for which the exopolysaccharide is as yet unknown (compare Figure 4 in this study to Figure 2 of Cao et al., 2017). Furthermore, the abundance of the PgaA secretin of PNAG biofilm exopolysaccharide is reduced in *X. nematophila* cells-lacking *nilB* and *nilC*, suggesting that the Δ*SR1* strain may be defective in PNAG exopolysaccharide biofilm formation. This change in PgaA abundance could be achieved through the RNA-binding protein CsrA (a carbon storage regulator), which is more abundant in Δ*SR1* relative to the SR1+ strain. In *E. coli*, CsrA is a global post-transcriptional regulator that coordinates diverse physiological processes, including iron storage and biofilm formation (Willias et al., 2015; Parker et al., 2017; Berndt et al., 2019; Pourciau et al., 2019) based on its negative regulation of PgaA translation (Wang et al., 2005; Figueroa-Bossi et al., 2014). In *Y. pestis*, biofilm formation is positively regulated by CsrA during growth on alternative carbon sources (Silva-Rohwer et al., 2021). Furthermore, in *Aggregatibacter actinomycetemcomitans*, CsrA-mediated carbon (glycogen) storage and peptidoglycan recycling are both modulated by the presence or absence of PgaA, indicating a complex system of feedback-signaling controlling flux of carbon through energy-deriving, storage, cell wall, and biofilm exopolysaccharide pathways (Shanmugam et al., 2015). Taken together, our data indicate that, as part of their function in nematode colonization, NilB and NilC are integrated with a complex system that balances growth, biofilm formation, carbon uptake and storage, and stress resistance pathways. Future work to identify the *X. nematophila* biofilm exopolysaccharides expressed at the nematode intestinal surface and the role, if any, of NilC in modulating their abundance should shed light on these questions.

A dual function for NilC in both the periplasm and extracellular space raises a further intriguing possibility that the TXISS_OMP_ NilB can control the abundance of NilC that exists in either orientation, perhaps modulated in response to different environmental conditions. Consistent with this idea, we found that, in *X. nematophila*, detectable surface exposure of NilC was observed only for strains in which *nilC* and *nilB* transcription is de-repressed by deletion of either of two transcription factors, NilR and Lrp. While the presence of NilB facilitates surface exposure of NilC during heterologous expression in *E. coli*, we noted a strong and direct correlation between total NilC levels and surface exposure. This phenomenon is similar to that observed for the *N. meningitidis* TXISS cargo protein fHbp. Low-level expression of some fHbp variants is tolerated, but overexpression induces surface exposure (da Silva et al., 2019). In these strains, the presence of the TXISS_OMP_ (Slam 1) is not necessary for, but does enhance the surface localization of fHbp (Fantappie et al., 2017). da Silva et al. (2019) found that the majority of *N. meningitidis* isolates express variant fHbp that is not secreted, suggesting that limiting surface exposure of fHbp likely confers a fitness benefit to the bacterium in a clinical setting (da Silva et al., 2019). Similarly, our finding that wild type *X. nematophila* has little detectable surface exposure of NilC indicates that there is selective pressure to limit surface exposure except under certain environmental conditions. Our finding that *X. nematophila*-expressing NilB variants with FLAG-tag insertions at the periplasmic N-terminus and in a surface-exposed loop display lower and higher NilC surface levels, respectively, relative to a cell-lacking detectable NilB (FLAG-379) indicates that this TXISS_OMP_ may regulate NilC surface exposure in response to periplasmic and extracellular signals.

Our overall working model (Figure 9) is that NilC secretion is triggered by the presence of extracellular glycan residues, potentially sensed by the TXISS_OMP_ NilB through one of its surface loop motifs (Bhasin et al., 2012). In the absence of such a glycan (which presumably indicates a non-nematode-host environment), NilC remains periplasmic and bound to peptidoglycan. In the presence of the activating extracellular glycan, TXISS-mediated secretion of NilC, either lipid-anchored in the outer leaflet, or threaded through TXISS_OMP_ NilB and lipid anchored in the inner membrane, facilitates binding or import of amino sugar molecules derived from the host cell surface. This model provides a framework for future studies, elucidating the identities and species specificity of nematode glycans and mucins, testing the ability of the TXISS_OMP_ NilB to recognize and respond to these host molecules by modulating secretion activity, establishing the structural orientation of NilC within the outer membrane, identifying the ligands of NilC N-terminal effector domains and determining the role, if any, of *Xenorhabdus*-derived PNAG or other exopolysaccharides on colonization of the *Steinernema* nematode anterior intestinal cecum.

## Materials and Methods

### Bacterial Strains and Media

Bacterial strains and plasmids used in this study are listed in Supplementary Table 1 and are described in more detail in the following sections. Bacteria were grown in lysogeny broth (LB) culture media (Miller, 1972) or minimal glucose media (Bhasin et al., 2012), supplemented with 1% LB at either 30°C (for *Xenorhabdus* strains) or 37°C (for *Escherichia coli* strains). For growth of *Xenorhabdus* strains, the LB medium was either stored in the dark (dark LB) or supplemented with 0.1% sodium pyruvate (Xu and Hurlbert, 1990). Strains of *X. nematophila* include those in which the SR1 region has been inserted into the *att*Tn*7* site, downstream of *glmS.* The Tn*7* transposon insertion introduces a new transcriptional terminator that results in 127-bp increase in total *glmS* transcript length (Gay et al., 1986). Previous studies in the closely related *E. coli* and *P. mirabilis* have not detected any detrimental effects from *att*Tn*7* insertion (Craig, 1996; Choi and Schweizer, 2006). Antibiotics were used as indicated at the following concentrations: ampicillin (Amp), 150 μg/ml; chloramphenicol (Cm), 15 μg/ml (*X. nematophila*) and 30 μg/ml (*E. coli)*; kanamycin (Kan), 50 μg/ml; erythromycin (Erm), 200 μg/ml; and streptomycin (7.5 μg/ml). All transformations were performed *via* electroporation at 2.5 kV for 6 milliseconds, followed by an hour of outgrowth at 37°C in SOC media.

### Generation of pETDuet-1 Expression Vectors

pETDuet-1 (Novagen ^®^) served as the plasmid backbone for cloning and expression. A 6xHis tag was added to the C-terminus of NilC *via* site-directed mutagenesis (using forward primer 5′-A GTCCATGGTAAATACAAAATCTAAAATCTATTTAGCTC-3′ and reverse primer 5′-GCTGCGGCCGCTTAGTGGTGGTG ATGATGATG-3′) prior to restriction cloning into MCS1 using the *Nco*I and *Not*I restriction sites to create pETDuet/MCS1:NilC Cterm6xHis. NilB-Flag26 was amplified from HGB1200 (Bhasin et al., 2012) and cloned into MCS2 *via* Gibson assembly (using two pairs of primers: NilB forward 5′-ATTAGTTAAGTATAAGAAGGAGATATACATATGAAAAA AATCAAATCCATCGTTATAAC-3′ and NilB reverse 5′-GCTC TCGAGTTAAAAATTACGCTTGAAGTCCAG-3′ plus pETDuet forward 5′-GACTTCAAGCGTAATTTTTAACTCGAGAGCTA ATTAACCTAGGCTGCTGCCAC-3′ and pETDuet reverse 5′-A TGTATATCTCCTTCTTATACTTAACTAATATACTAAGATG G-3′) to create the plasmid pETDuet/MCS2:NilB-FLAG-26/MCS1:NilC_Cterm6xHis. Plasmids were transformed into *E. coli* DE3 C43 (Lucigen ^®^) *via* electroporation prior to expression. *nilB* and *nilC* sequences were confirmed by Sanger sequencing at the University of Tennessee (UT) Genomics Core. Expression of NilB and NilC was confirmed *via* immunoblotting of induced lysates using α-FLAG and α-NilC antibodies, respectively.

### Measuring NilC Surface Exposure in *Escherichia coli* Co-expression Strains by Immuno-Dot Blotting

*Escherichia coli* BL21 DE3 C43 strains containing pETDuet-1/MCS2:NilB_26aaflag/MCS1:NilC_Cterm6xHis, pETDuet-1/MCS1:NilC_Cterm6xHis, and no plasmid (HGB 2534, 2535, and 2536, respectively) were raised on defined glucose medium (Orchard and Goodrich-Blair, 2004) plates at 37°C for ∼16 h. HGB 2534 and 2535 plates were supplemented with ampicillin at 150 μg/ml. Biological replicates (10 each) of HGB 2534 and 2535 were cultured into 5 ml of a defined glucose medium supplemented with 1% LB (Amp 150 μg/ml) and incubated at 37°C for ∼16 h. HGB 2536 was cultured in biological triplicate into 5 ml of a defined glucose medium supplemented with 1% LB. Overnight cultures were rinsed 2 x in sterile-defined media, and then half was aliquoted into 5-ml fresh defined glucose media supplemented with 1% LB, and half was aliquoted into 5-ml fresh dark LB. These cultures were incubated at 37°C for 3 h prior to induction with 0.5-mM IPTG. Cultures were induced at 37°C for 2 h. Cultures were equalized to an OD_600_ of 0.6 and rinsed 3 x in Phosphate Buffered Saline (PBS). Note that, even for those cultures that did not grow, sufficient cells were present to allow spotting of the normalized OD amount. For each whole cell sample, 2 μl was spotted onto a nitrocellulose membrane and allowed to dry. Cells were lysed by sonication (30 s at ∼500-rms volts), and 2 μl of each lysate sample was spotted onto a nitrocellulose membrane and allowed to dry. Membranes were immunoblotted and enumerated as described under “Measuring *X. nematophila* NilC surface exposure” below.

### Construction of *Xenorhabdus nematophila* Mutants-Expressing Modified NilC

Site-directed mutagenesis of pTn7/SR1 (Cowles and Goodrich-Blair, 2004) was used to generate *nilC*57-V17A-C20S wherein the lipobox was removed by adding a signal peptidase I site and by removing the conserved lipobox cysteine residue (using forward primer 5′-GCCTCTTCTAGAGGAGGGGGTTCT-3′ and reverse primer 5′-TGTAGCAGAAAGTACCAGTGCGAG-3′). Site-directed mutagenesis of pAB001 (Bhasin, Chaston, Goodrich-Blair) and pTn7/SR1-*nilC*57-V17A-C20S was used to generate *nilC*58-6xHis and *nilC*59-V17A-C20S-6xHis wherein 6 histidine residues were encoded onto the C-terminal end of *nilC* (using forward primer 5′-AGTCC ATGGTAAATACAAAATCTAAAATCTATTTAGCTC-3′ and reverse primer 5′-GCTGCGGCCGCTTAGTGGTGGTGATGAT GATG-3′). The three resulting plasmids were transformed into *E. coli* BW29427, a DAP-dependent, conjugation proficient strain, resulting in HGB2308, HGB2309, and HGB2321. Triparental conjugation was performed using an *X. nematophila* background (either HGB0777 or HGB1251), the appropriate pTn*7* plasmid, and the helper plasmid pUX-BF13 (HGB0283) at a ratio of 3:1:1. Exconjugants were selected for using kanamycin resistance, and insertion into the *att*Tn7 site was confirmed *via* Sanger sequencing (using forward primer 5′-TGTTGGTTTCACATCC-3′ and reverse primer 5′-TACTTATGAGCAAGTATTGTC-3′), resulting in HGB2330, HGB2331, HGB2332, HGB2371, HGB2372, and HGB2373. GFP-expressing strains possessing the modified *nilC* alleles were constructed by conjugating *X. nematophila* stains HGB2330, HGB2331, and HGB2332 and *E. coli* S17-1 λpir-containing pJMC001 (HGB1783) at a ratio of 3:2. Exconjugants were selected for using chloramphenicol resistance and screened for GFP expression using fluorescence microscopy, resulting in HGB2368, HGB2369, and HGB 2370.

### Infective Juvenile Colonization Assays

To prepare nematodes for colonization assays, nematode eggs were collected from gravid adult females, developing from infective juvenile nematodes inoculated onto wild type lawns of symbiont and surface sterilized as previously described (Murfin et al., 2012). They were then applied in biological triplicate to LA plates with lawns of treatment bacteria (HGB800, HGB2330, HGB2331, HGB2332, HGB2106, HGB2368, HGB2369, and HGB2370; see Supplementary Table 1). Lawns were incubated at 27°C for 9 days and then transferred to white traps to collect emerging infective juveniles. Nematodes were collected and stored in sterile water at 27°C in tissue culture flasks. Frequency of colonization was determined *via* fluorescence microscopy on a Keyence BZX-700. Briefly, nematodes, which had been raised on the GFP-expressing bacterial strains, were observed for intestinal GFP presence at 4 and 20 days post emergence. Both time points gave similar results, suggesting that no mutant suffered from a persistence defect. Direct counts of average CFU/nematode were determined *via* a grinding assay wherein nematodes that had been raised on non-fluorescent bacterial strains were collected 6 days post emergence, equalized by density, homogenized *via* an electric mortar and pestle, serially diluted, and plated on LB plates to determine CFU. Dilutions of homogenate equivalent to 4 and 0.4 nematodes were used for quantification.

### Expression, Purification, and Biophysical Characterization of the NilC Soluble Domain

#### Expression and Purification of the NilC Soluble Domain

NilC lacking its predicted signal peptide (NilC amino acids 22-282) was cloned by GenScript into the vector pET-28a(+)-TEV using restriction enzymes Nde1 and Not1. The resulting ORF includes an N-terminal hexahistidine tag, followed by a Tobacco Etch Virus protease (TEV) cleavage site. Three amino acids from the cloning (GHM) remain prepended to the NilC sequence. NilC_22–282_ was expressed in *E. coli* BL21(DE3) cells. The NilC_62–282_ construct was prepared using pET-28a(+)-TEV-NilC22-282 as a template where the sequence for residues 22–61 was deleted by inverse PCR with the following primers: forward: 5′-CTTAAGGGATATTCCAACG-3′, reverse: 5′-CATATGGCCCTGAAAATAAAG-3′. The PCR product was treated with a Dpn1 restriction enzyme for 3 h at 37°C, and then phosphorylated with T4 DNA kinase, followed by ligation with T4 DNA ligase at 16°C overnight. Since this construct is based on pET-28A(+) TEV NilC22-282 construct, it has no signal peptide, so it is expressed in the cytoplasm.

For expression, cells were grown at 37°C in LB media until OD_600_ reached ∼0.7, at which point expression was induced by adding IPTG to a final concentration of 1 mm. Protein expression was performed at 25°C overnight. For isotopically labeled protein for NMR experiments, cells were again grown in LB but then transferred to M9 minimal media, supplemented with ^15^N ammonium chloride (Cambridge Isotope Laboratories) (1g/L of culture). Protein induction and expression were performed as above.

For purification, the cell pellet was resuspended in Buffer A (50 mM Tris-HCl pH 8.0, containing 300-mM NaCl). Cell lysis was preceded by French press. Cell lysate was clarified by centrifugation at 25,000 × g for 30 min at 8°C before being loaded into a 5-ml Ni-NTA column (Qiagen, Germantown, MD, United States). After washing with 100-ml of Buffer A containing 50-mM imidazole, protein was eluted using Buffer A with 500-mM Imidazole. Fractions with target protein were pooled and mixed with TEV protease (final concentration of 1 mg/ml), removing the hexahistidine tag. The sample was dialyzed against Buffer A overnight before removal of the TEV protease using a 1-ml Ni-NTA column. Fractions containing NilC were concentrated before loading into a Sephacryl S-100 HiPrep 16/60 column (Cytiva) for size exclusion chromatography. This step was performed in 50-mM Tris-HCl pH 8.0, containing 150-mM NaCl.

#### Solution NMR

2D ^1^H-^15^N HSQC was performed in a 750-MHz NMR spectrometer equipped with a cryoprobe at the National Magnetic Resonance Facility at Madison. A NilC sample was prepared in 25-mM sodium phosphate buffer pH 6.5, containing 25-mM NaCl, 0.01% sodium azide, 50-μM 2,2-dimethyl-2-silapentane-5-sulfonate (DSS), and 8% D_2_O. Experiments were performed at 27°C using a 180-μM NilC concentration. Spectra were processed using Bruker Topspin and referenced to a DSS standard.

#### Circular Dichroism Spectroscopy

Circular dichroism spectra were collected in an AVIV model 420 CD spectrometer. NilC was prepared at 0.092 mg/ml in a 25-mM sodium phosphate buffer, pH 6.5. Data acquisition was performed at 25°C in a 1-mm path length cuvette using a 5-s averaging time. The CD spectrum of a buffer was measured and subtracted from the NilC spectrum. Secondary structure calculation was performed using the programs Bestsel (Micsonai et al., 2015) and CONTIN/LL (Provencher and Glockner, 1981; van Stokkum et al., 1990).

#### Limited Proteolysis

Purified NilC at 1 mg/ml (35 mm) was mixed with proteinase K (PK) in a 100:1 molar ratio in 100 ml. The reaction was treated after 1 or 5 min using the following methods: (1) no treatment (2) heating at 95°C for 10 min (3) addition of PMSF to 1-mM final concentration (4) addition of a protease inhibitor cocktail (Roche) to 1 X final concentration. Each sample was then diluted with an SDS-PAGE sample buffer and heated at 95°C for 10 min. All samples were left at 4°C for ∼1 week before running SDS-PAGE. The mass of the major breakdown product was determined by an Electrospray Ionization Mass Spectrometry run in the positive mode. To determine which structural element of NilC this corresponded to, we used both PeptideCutter and MassPeptide (which consider ion charges) to predict all of the possible Proteinase K digestion peptides and their masses, and a simple spread sheet to sum the masses of all possible contiguous peptides to identify those which could be the observed stable fragment.

HGB1211 (*X. nematophila* with *nilR* and SR1 deleted and the SR1 locus carrying a *nilB*-FLAG26 insertion and a *nilC* (M1Z) start-to-stop codon mutation introduced at the *att*Tn*7* locus) were created as described previously (Cowles and Goodrich-Blair, 2004; Bhasin et al., 2012). Briefly, the plasmid pAB001 (Bhasin et al., 2012) (pTn*7*-SR1 *nilB*-FLAG26) was used as a template for site-directed mutagenesis with primers NilCMtoZfor 5′-CAAATTGGAATCATTATTAGAATACAAAATCTAAAATC-3′ and NilCMtoZrev 5′-TTTAGATTTTGTATTCTAATAATGATT CCAATTTGTTT-3′ (Cowles and Goodrich-Blair, 2004). The resulting plasmid, pEVS107:SR1/*nilB-*FLAG26; *nilC* (M1Z), was conjugated into *X. nematophila*Δ*nilR16:Str*Δ*SR1-7:kan.*

### Measuring *Xenorhabdus nematophila* NilC Surface Exposure by Immuno-Dot Blotting and Flow Cytometry

NilC surface exposure in *X. nematophila* cells was monitored by immuno-dot blotting of whole bacterial cells. For Figures 3A,B, these were HGB800, HGB1102, HGB778, and HGB779; for Figures 3C,D, these were HGB1966, 1967, and 1968; for Figures 3E,F, these were HGB1200, HGB1211, HGB1207, and HGB1808 (see Supplementary Table 1). For Supplementary Figure 10, strains tested were HGB800, 1103, 2330, 2371 (see Supplementary Table 1). Test strains were struck on LB pyruvate plates with appropriate antibiotics and incubated at 30°C overnight. For each strain, 5 ml of LB that was kept in the dark to prevent generation of reactive oxygen species (dark LB) was inoculated and grown shaking overnight at 30°C. Strains were then sub-cultured 1:1,000 in dark LB and grown overnight (∼16 h) at 30°C with shaking. After growth, cells were spun down from the media and rinsed two times in 1xPBS. Where appropriate, the supernatants were collected and sterilized using a 0.22-mm filter to generate cell-free supernatants. Cell concentrations were normalized to an OD_600_ of 6 and serially diluted 1:3 two times, giving OD_600_ values of 6, 2, and 0.67. Each dilution of whole cells was then spotted in technical triplicate onto a PVDF or a nitrocellulose membrane in 2-μl aliquots and allowed to dry. Lysates were obtained by adding glass beads and shaking vigorously using a Fastprep homogenizer in three cycles of 20 s, shaking, 5 min waiting at 4°C. Lysed cells were centrifuged at 15,871 × *g* for 5 min to remove cellular debris. Lysates and cell-free supernatants were spotted onto nitrocellulose as above allowed to dry. Membranes were blocked with a 50:50 mixture of a Li-cor Intercept Protein-Free Blocking Buffer (P/N: 927-80001) and Tris buffered saline (TBS: 50-mM Tris-Cl, pH 7.6; 150-mM NaCl) for 1 h. Membranes were incubated in a 50:50 Li-cor blocking buffer: TBS supplemented with 0.1% Tween 20 and a 1:1,500 Rabbit Anti-NilC antibody (Pocono labs, received July 23, 2019) for 1 h. Membranes were rinsed 4 times in TBS supplemented with 0.1% Tween 20 prior to incubation with a 1:5,000 Goat anti-rabbit secondary antibody bound to an IRDye 680RD fluorophore. Emission intensity at 700 nm was quantified using an Odyssey Infrared Imaging System and statistically analyzed *via* one-way ANOVA with Tukey’s *post hoc* multiple comparisons analysis.

For flow cytometry, test strains were struck on LB pyruvate plates with appropriate antibiotics and grown 30°C for 1–2 days. Cultures of 5 ml of a minimal medium (MM) (Bhasin et al., 2012), supplemented with 0.1% casamino acids with no antibiotics, were inoculated with three biological replicates for each test strain and grown 30°C overnight shaking to a stationary phase. Cells were pelleted by centrifugation at 3,220 × *g* for 5 min and resuspended in 1 ml of phosphate buffered saline (1X PBS:0.137-M NaCl, 0.0027-M KCl, 0.01-M Na_2_HPO_4_, 0.0018-M KH_2_PO_4_). Each biological replicate was divided into two treatment groups, one with permeabilization (0.1% TritonX100 in 1X PBS final concentration), and one without permeabilization (PBS) and incubated at 25°C shaking for 10 min. All remaining centrifugation was performed at 6,010 × *g* for 1 min. Samples were washed in 500-μl 1X PBS, and the primary α-NilC IgG antibody (Cowles and Goodrich-Blair, 2004) was added at 1:200 concentration, and incubated at 25°C shaking for 1 h. The samples were washed again in PBS and the secondary goat α-rabbit IgG antibody was added at 1:200 and incubated at 25°C shaking for 1 h. The samples were washed a final time in PBS, and then measured for fluorescence at 488 nm using a BD Biosciences LSR flow cytometer. HGB2018 expressing GFP was used as a 488nm positive control. The gate for positive signal at 488nm was set at 97% of the HGB2018 cells.

### Lectin Localization and Bacterial Colonization Competition Assays

#### Nematode Strains and Preparation

*Steinernema carpocapsae* ALL strain was obtained from Dr. Harry Kaya (UC-Davis). *S. feltiae* was obtained from the laboratories of Dr. S. Patricia Stock (U Arizona) and was originally isolated in FL, United States. Each of these nematodes is propagated approximately every 3 months through *Galleria mellonella* insects (Vivas and Goodrich-Blair, 2001). *S. scapterisci* was obtained from Becker Underwood Inc. and is maintained by propagation approximately every 3 months through *Acheta domesticus* crickets obtained from local pet stores (Kim et al., 2017). Infective juvenile stage nematodes are stored in water in 50-ml tissue culture flasks in between insect infections.

#### Lectin Binding Assays

To prepare nematodes for lectin binding and colonization assays, nematode eggs were collected from gravid adult females, developing from infective juvenile nematodes inoculated onto wild type lawns of symbiont and surface sterilized as previously described (Murfin et al., 2012). They were then applied to treatment plates (e.g., bacterial symbiont or liver kidney agar, which enables nematode development without a bacterial symbiont; Martens et al., 2005) as detailed for each experiment. If nematode eggs were not immediately seeded onto treatment plates, they were stored in a sealed dish in dark LB for up to 4 days. After the eggs had been placed on the plates, they were stored at 27°C in the dark for 9 days. Every 3 days, a fresh plate was selected from each treatment and the nematodes collected off the plates by washing and pipetting with 1x PBS. Collected nematodes were placed in a 1.5-ml centrifuge tube and rinsed three additional times with 1x PBS. After rinsing, as much liquid was removed from the tube and 200 μL of 1x PBS added back to equalize the volume within each tube. For treatment with lectin, a final concentration of 27 mM (∼2 μL) of a green fluorescein conjugate lectin was added to the nematode preparation. Tubes were wrapped in foil to inhibit light bleaching and shaken gently overnight on an end-over-end mixer. Nematodes were then rinsed three times with 1X PBS to remove excess lectin before observation *via* fluorescence microscopy on a Keyence BZX-700 microscope. For all data shown in Figures 4, 5, experimental observations of lectin or bacterial localization were blinded, such that the observer was not aware of the treatment. For Figure 4A, *S. carpocapsae* nematodes were stained with 6.6-mM rhodamine phalloidin (Sigma) as described in Chaston et al. (2013) and visualized on a Zeiss LSM 510 confocal microscope (Zeiss, Thornwood, NY, United States) (Sugar et al., 2012). Images for Figures 4B,D were taken on a Nikon Eclipse TE300 epifluorescence-inverted microscope. Brightfield and FITC filter images were overlaid and false colored using Image-J.

#### NilC Protein and Unconjugated Ulex Europaeus Agglutinin and Wheat Germ Agglutinin Lectin Colonization Competition Assays

*Xenorhabdus nematophila* HGB2018, *X. innexi* HGB2171, and *X. bovienii* HGB1865 bacteria expressing the green fluorescent protein from the *att*Tn*7* locus were used to visualize bacteria at the anterior intestinal cecum colonization site of *S. carpocapsae, S. scapterisci*, and *S. feltiae* FL nematodes, respectively, in the presence or absence of lectin or NilC protein. In addition, to determine the effects of WGA on the colonization of *X. nematophila* with and without SR1, isogenic Δ*SR1* strains with either empty Tn*7* or the Tn*7-*SR1 locus were compared. The green-fluorescent protein was introduced into these strains by integrating the plasmid pJMC001 at the *kefA* site (Bhasin et al., 2012). Nematodes isolated as described above were exposed to GFP-expressing bacterial symbionts alone or with unconjugated WGA, unconjugated UEA, or purified NilC (see above) at 27-mM final concentration, and processed as described above before observing by fluorescence microscopy for the presence or absence of bacteria at the AIC. As above, these experiments were blinded.

#### Statistical Analysis

Separate one-way analyses of variance were performed in GraphPad Prism to test if treatments differed with respect to mean fluorescence intensity. Tukey’s *post hoc* multiple comparisons analysis was used to compare between all levels of treatment. A simple linear regression was used to test if total NilC expression predicted surface exposure of NilC.

For WGA, UEA, and soluble NilC protein experiments related to individual nematode colonization, the binary outcome of colonization was analyzed within generalized linear mixed models using the GLIMMIX procedure (SAS v 9.4, Cary, NC, United States) (Schabenberger, 2005) with a binary distribution and a logit link. The fixed effect of treatment, as well as other confounding variables, such as days observed (6 days total) or the life stage of the nematode (male, female, juvenile), were included in the model. Additionally, random effects to account for biological replicate were included. The *inverse link* option was used within the LS Means statement to obtain model-adjusted probability for colonization by bacterial symbionts. Additional variables, including addition of unconjugated WGA and bacteria, or bacteria alone, the life stage of the nematode, and the day observed that can contribute to colonization status in the assay, were included in models as appropriate. Statistical significance was set at α = 0.05.

### Proteomics and Metabolomics

#### Sample Preparation

HGB 1495 (*X. nematophila* ATCC19061 Δ*SR1*Δ*nilR* GFP with integrated empty Tn*7*) and HGB1496 (*X. nematophila* ATCC19061 Δ*SR1*Δ*nilR*GFP with integrated Tn*7* containing SR1) were struck from frozen stocks onto LB pyruvate plates and incubated at 30°C overnight. Three biological replicates for each strain were inoculated in 50 ml of a minimal medium (Bhasin et al., 2012) in a sterile 500-ml flask, and incubated at 30°C, 200 rpm to OD_600_ of 0.6. Based on the findings of Bhasin et al. (2012), growth in this medium to OD_600_ of 0.6 for ∼55 h was expected to maximize *nilB* expression in the HGB1496 strain. Cell lysis was monitored at 260–280 Abs. The 50-ml culture was divided for the metabolomics and proteomics analyses. For proteomic analysis, cell pellets were suspended in an SDS lysis buffer (2% in 100 mM of NH4HCO3, 10-mM DTT). Samples were physically disrupted by bead beating (0.15 mm) at 8,000 rpm for 5 min. Crude lysates were boiled for 5 min at 90°C. Cysteines were blocked by adjusting each sample to 30-mM iodoacetamide and incubating the reaction in the dark for 15 min at room temperature. Proteins were precipitated using a chloroform/methanol/water extraction. Dried protein pellets were resuspended in 2% sodium deoxycholate (SDC) (100-mM NH_4_HCO_3_), and protein amounts were estimated by performing a bicinchoninic acid (BCA) assay. For each sample, an aliquot of ∼500 μg of protein was digested *via* two aliquots of sequencing-grade trypsin [Promega, 1:75 (w:w)] at two different sample dilutions, (overnight) and subsequent incubation for 3 h at 37°C. The peptide mixture was adjusted to 0.5% formic acid to precipitate SDC. Hydrated ethyl acetate was added to each sample at a 1:1 [v:v] ratio three times to effectively remove SDC. The samples were then placed in a SpeedVac Concentrator (Thermo Fisher Scientific, Waltham, MA, United States) to remove ethyl acetate and further concentrate the sample. The peptide-enriched flowthrough was quantified by a BCA assay, desalted on RPC18 stage tips (Pierce Biotechnology, Waltham, MA, United States) and then stored at −80°C. For metabolomic analysis, frozen samples were thawed at 4°C prior to extraction. Extractions were performed using 1.5 ml of 0.1-M formic acid in 4:4:2 acetonitrile:water:methanol according to the procedure described previously (Dearth et al., 2018).

#### LC-MS/MS and UPLC-HRMS Analysis

For proteomics analyses, all samples were analyzed on a Q Exactive Plus mass spectrometer (Thermo Fisher Scientific, Waltham, MA, United States), coupled with a Proxeon EASY-nLC 1200 liquid chromatography (LC) pump (Thermo Fisher Scientific, Waltham, MA, United States). Peptides were separated on a 75-μm inner diameter microcapillary column packed with 25 cm of Kinetex C18 resin (1.7 μm, 100 Å, Phenomenex). For each sample, a 2 μg aliquot was loaded in Buffer A (0.1% formic acid, 2% acetonitrile) and eluted with a linear 150-min gradient of 2–20% of Buffer B (0.1% formic acid, 80% acetonitrile), followed by an increase in Buffer B to 30% for 10 min, another increase to a 50% buffer for 10 min and concluding with a 10-min wash at 98% Buffer A. The flow rate was kept at 200 nl/min. MS data were acquired with the Thermo Xcalibur software version 4.27.19, a topN method where N could be up to 15. Target values for the full scan MS spectra were 1 × 106 charges in the 300–1,500 m/z range with a maximum injection time of 25 ms. Transient times corresponding to a resolution of 70,000 at *m/z* 200 were chosen. A 1.6 *m/z* isolation window and fragmentation of precursor ions were performed by higher-energy C-trap dissociation (HCD) with a normalized collision energy of 30 eV. MS/MS scans were performed at a resolution of 17,500 at m/z 200 with an ion target value of 1 × 106 and a maximum injection time of 50 ms. Dynamic exclusion was set to 45 s to avoid repeated sequencing of peptides.

An established untargeted metabolomics method utilizing ultra-high-performance liquid chromatography, coupled to high-resolution mass spectrometry (UHPLC-HRMS) (Thermo Scientific, San Jose, CA, United States), was used to analyze water-soluble metabolites (Metabolomic Analysis *via* Reversed-Phase Ion-Pairing Liquid Chromatography Coupled to a Stand-alone Orbitrap Mass Spectrometer). Synergi 2.6-μm Hydro RP column 100 Å, 100 mm × 2.1 mm (Phenomenex, Torrance, CA, United States) and an UltiMate 3000 pump (Thermo Fisher Scientific, Waltham, MA, United States) were used to carry out the chromatographic separations prior to full scan mass analysis by an Exactive Plus Orbitrap MS (Thermo Fisher Scientific, Waltham, MA, United States). HPLC grade solvents (Thermo Fisher Scientific, Waltham, MA, United States) were used. Chromatographic peak areas for each detected metabolite were integrated using an open-source software package, Metabolomic Analysis and Visualization Engine (MAVEN). Area under the curve (AUC) was used for further analyses. The raw metabolomics data have been submitted to the MetaboLights data repository under study ID MTBLS3857.

#### Proteome Database Search Analysis

MS raw data files were searched against the predicted proteins of the *X. nematophila* ATCC 19061 genome (accession FN667742; downloaded 12/20/2017) (Chaston et al., 2013) to which common contaminant proteins had been added. A decoy database, consisting of the reversed sequences of the target database, was appended in order to discern the false-discovery rate (FDR) at the spectral level. For standard database searching, the peptide fragmentation spectra (MS/MS) were analyzed by the Crux pipeline v3.0. The MS/MS was searched using the Tide algorithm and was configured to derive fully tryptic peptides using default settings, except for the following parameters: allowed clip nterm-methionine, precursor mass tolerance of 10 parts per million (ppm), a static modification on cysteines (iodoacetamide; + 57.0214 Da), and dynamic modifications on methionine (oxidation; 15.9949). The results were processed by Percolator to estimate q values. Peptide spectrum matches (PSMs) and peptides were considered to be identified at a *q* value < 0.01. Across the entire experimental dataset, proteins were required to have at least 2 distinct peptide sequences and 2 minimum spectra per protein. For label-free quantification, MS1-level precursor intensities were derived from MOFF using the following parameters: 10 ppm mass tolerance, a retention time window for extracted ion chromatogram was 3 min, a time window to get the apex for the MS/MS precursor was 30 s. Protein intensity-based values, which were calculated by summing together quantified peptides, normalized by dividing by protein length and then LOESS and median central tendency procedures, were performed on log_2_-transformed. Using the freely available software Perseus^1^, missing values were replaced by random numbers drawn from a normal distribution (width = 0.3 and downshift = 2.8). This platform was also used to generate the volcano plots.

### Peptidoglycan-Binding Assays

NilC was purified as described above in “Expression and purification of the NilC soluble domain.” Bovine serum albumin (BSA) was purchased from Fisher Scientific (BP9700100) and used as a negative control for peptidoglycan binding. Peptidoglycan (PG) from *Bacillus subtills* (SMB00288) and chitin (C7170-100G) were purchased from Sigma Aldrich. All reactions were performed in a protein storage buffer (50-mM Tris HCL, 500-mM NaCl pH8). In 50-μl binding reactions, 1 μg of either pure NilC or BSA was combined with 500, 200, 20, or 0 μg of PG or chitin. All reactions were incubated, rotating for 1 h at 37°C. After incubation, reactions were centrifuged at 21,000 G for 3 min to pellet the insoluble fraction and collect the supernatant. This process was repeated two times to ensure complete removal of insoluble components. The remaining pellet was rinsed three times with a protein storage buffer. About 2 μl of each supernatant sample and a pellet sample was dot blotted in technical triplicate onto nitrocellulose membranes. Membranes were heat fixed at 50°C for 10 min to adhere the PG. Membranes were blocked with a 50:50 mixture of Li-cor Intercept Protein-Free Blocking Buffer (P/N: 927-80001) and Tris buffered saline (TBS: 50-mM Tris-Cl, pH 7.6; 150-mM NaCl) for 1 h. NilC membranes were immunoblotted and enumerated as described under “Measuring *X. nematophila* NilC surface exposure” above. BSA membranes were incubated in a 50:50 Li-cor blocking buffer, TBS supplemented with 0.1% Tween 20 and 1:5,000 rabbit anti-BSA IgG [Thermo Fisher Scientific (Waltham, MA, United States), A11133] for 1 h. The membranes were rinsed four times in TBS supplemented with 0.1% Tween 20 prior to incubation with 1:5,000 a Goat anti-rabbit secondary antibody bound to an IRDye 680RD fluorophore. For all membranes, emission intensity at 700 nm was quantified using an Odyssey Infrared Imaging System. A Tukey’s honest significance test was used to assess differences between NilC and BSA intensities; a Dunnett’s test was used to assess differences between PG binding and chitin binding for each concentration.

## Data Availability Statement

The datasets presented in this study can be found in online repositories. The names of the repository/repositories and accession number(s) can be found below: MetaboLights data repository under study ID MTBLS3857.

## Author Contributions

HG-B and KF contributed to conception and design of the study, contributed to data analysis, and wrote the first draft of the manuscript. AG, CE, EM, NM, and TM designed and performed experiments, analyzed results, and wrote sections of the manuscript. CM and KJ performed experiments and analyzed results. LS performed statistical analyses. PA, RH, and SC contributed to experimental design and analysis. All authors contributed to manuscript revision, read, and approved the submitted version.

## Conflict of Interest

The authors declare that the research was conducted in the absence of any commercial or financial relationships that could be construed as a potential conflict of interest.

## Publisher’s Note

All claims expressed in this article are solely those of the authors and do not necessarily represent those of their affiliated organizations, or those of the publisher, the editors and the reviewers. Any product that may be evaluated in this article, or claim that may be made by its manufacturer, is not guaranteed or endorsed by the publisher.

## Footnotes

1
https://maxquant.net/perseus/
