# Supplementary material for: A Surface Exposed, Two-Domain Lipoprotein Cargo of a Type XI Secretion System Promotes Colonization of Host Intestinal Epithelia Expressing Glycans
Source: Front Microbiol. 2022 Apr 29;13:800366. doi: 10.3389/fmicb.2022.800366 (PMC9100927; doi:10.3389/fmicb.2022.800366)
Supplement: Supplementary file 3 [file Data_Sheet_3.pdf]

Supplementary Material

A Surface Exposed, Two-Domain Lipoprotein Cargo of a Type XI Secretion System Promotes Colonization of Host Intestinal Epithelia Expressing Glycans

Alex S. Grossman[1¶], Cristian A. Escobar[2¶], Erin J. Mans[1¶], Nicholas C. Mucci[1], Terra J. Mauer[2], Katarina A. Jones[3], Cameron C. Moore[1], Paul E. Abraham[4], Robert L. Hettich[4], Liesel Schneider[5], Shawn R. Campagna[4,6], Katrina T. Forest[2*], and Heidi Goodrich-Blair[1,2*]

[1]University of Tennessee-Knoxville, Department of Microbiology, Knoxville, TN
[2]University of Wisconsin-Madison, Department of Bacteriology, Madison, WI, 53706
[3]Department of Chemistry, University of Tennessee-Knoxville, Knoxville, TN 37996, USA
[4]Biosciences Division, Oak Ridge National Laboratory, Oak Ridge TN, USA
[5]Department of Animal Sciences, University of Tennessee-Knoxville, Knoxville, TN 37996, USA
[6]Biological and Small Molecule Mass Spectrometry Core, University of Tennessee-Knoxville, Knoxville, TN 37996, USA
¶These authors contributed equally
*Co-corresponding authors

Table 1: Bacterial Strains and Plasmids used in this Study

Strain	Genotype	Plasmid	Antibiotic Resistance	Reference/Source
HGB2534	*Escherichia coli* BL21 DE3 C43	pETDuet/MCS2:NilB-FLAG-26/MCS1:NilC_Cterm6xHis	Amp	This Study
HGB2535	*Escherichia coli* BL21 DE3 C43	pETDuet/MCS1:NilC Cterm6xHis	Amp	This Study
HGB2536	*Escherichia coli* BL21 DE3 C43	NA	NA	(Kwon et al., 2015)
NA	*Escherichia coli* BL21 DE3	pET-28a(+)-TEV-*nilC*$_{22\text{-}282}$	Kan	This Study; Genscript
HGB1783	*Escherichia coli* S17-1 λpir	pJMC001	Cm	(Bhasin et al., 2012)
HGB0283	*Escherichia coli* S17-1 λpir	pUX-BF13 Tn*7* transposition helper	Amp	(Bao et al., 1991)
HGB2308	*Escherichia coli* BW29427 (DAP-requiring)	pTn*7*/SR1- *nilC57*-V17A-C20S	Kan, Strep, Erm	This Study
HGB2309	*Escherichia coli* BW29427 (DAP-requiring)	pTn*7*/SR1-*nilB26*-FLAG26 *nilC58*-6XHis	Kan, Strep, Erm	This Study
HGB2321	*Escherichia coli* BW29427 (DAP-requiring)	pTn*7*/SR1- *nilC59*-V17A-C20S-6XHis	Kan, Strep, Erm	This Study
HGB2018	*Xenorhabdus nematophila* HGB800 *att*Tn*7*::Tn*7*/GFP	NA	Amp, Cm	Lab strain; (Sugar et al., 2012)
HGB2106	*Xenorhabdus nematophila* HGB800 *kefA*::pJMC001-GFP (from HGB1783)	NA	Amp, Cm	This Study
HGB2330	*Xenorhabdus nematophila* Δ*SR1-7::kan* *att*Tn*7*::Tn*7*/SR1-*nilC57*-V17A-C20S	NA	Amp, Kan, Erm	This Study
HGB2331	*Xenorhabdus nematophila* Δ*SR1-7::kan* *att*Tn*7*::Tn*7*/SR1-*nilB26*-FLAG26 *nilC58*-6XHis	NA	Amp, Kan, Erm	This Study
HGB2332	*Xenorhabdus nematophila* Δ*SR1-7::kan* *att*Tn*7*::Tn*7*/SR1- *nilC59*-V17A-C20S-6XHis	NA	Amp, Kan, Erm	This Study
HGB2368	*Xenorhabdus nematophila* Δ*SR1-7::kan* *att*Tn*7*::Tn*7*/SR1-*nilC57*-V17A-C20S *kefA*::pJMC001-GFP (from HGB1783)	NA	Amp, Kan, Erm, Cm	This Study
HGB2369	*Xenorhabdus nematophila* Δ*SR1-7::kan* *att*Tn*7*::Tn*7*/SR1-*nilB26*-FLAG26 *nilC58*-6XHis *kefA*::pJMC001-GFP (from HGB1783)	NA	Amp, Kan, Erm, Cm	This Study
HGB2370	*Xenorhabdus nematophila* Δ*SR1-7::kan* *att*Tn*7*::Tn*7*/SR1-*nilC59*-V17A-C20S-6XHis *kefA*::pJMC001-GFP (from HGB1783)	NA	Amp, Kan, Erm, Cm	This Study
HGB2371	*Xenorhabdus nematophila* Δ*nilR16::Sm* Δ*SR1-7::kan* *att*Tn*7*::Tn*7*/SR1-*nilC57*-V17A-C20S	NA	Amp, Kan, Erm, Str	This Study
HGB2372	*Xenorhabdus nematophila* Δ*nilR16::Sm* Δ*SR1-7::kan*	NA	Amp, Kan, Erm, Str	This Study

	*att*Tn*7*::Tn*7*/SR1-*nilB26*-FLAG26 *nilC58*-6XHis			
HGB2373	*Xenorhabdus nematophila* Δ*nilR16::Sm* Δ*SR1-7::kan* *att*Tn*7*::Tn*7*/SR1-*nilC59*-V17A-C20S-6XHis	NA	Amp, Kan, Erm, Str	This Study
HGB800	*Xenorhabdus nematophila* WT isolated from *Steinernema carpocapsae* nematodes	NA	Amp	ATCC 19061 (Chaston et al., 2011)
HGB1102	*Xenorhabdus nematophila* HGB800 Δ*nilR16::Strep*	NA	Str	(Cowles and Goodrich-Blair, 2006)
HGB1103	*Xenorhabdus nematophila* Δ*nilR16::Sm*	NA	Amp, Str	(Cowles and Goodrich-Blair, 2006)
HGB1255	*Xenorhabdus nematophila* Δ*nilR16::Sm* Δ*SR1-7::kan* *att*Tn*7*::Tn*7*-SR1	NA	Amp, Kan, Erm, Str	(Bhasin et al., 2012)
HGB1103	*Xenorhabdus nematophila* Δ*nilR16::Sm*	NA	Amp, Str	(Cowles and Goodrich-Blair, 2006)
HGB1966	*X. nematophila lrp-2::kan*	pKV69 (vector)	Cm	(Hussa et al., 2015)
HGB1967	*X. nematophila lrp-2::kan*	pEH54 (low-Lrp plasmid)	Cm	(Hussa et al., 2015)
HGB1968	*X. nematophila lrp-2::kan*	pEH56 (high-Lrp plasmid)	Cm	(Hussa et al., 2015)
HGB1521	*Escherichia coli* S17-1 λpir	pEVS107::SR1/*nilB*-FLAG26; *nilC*(M1Z)	Kan, Erm, Str, Spec	This Study
HGB1200	*Xenorhabdus nematophila* Δ*nilR16::Str* Δ*SR1-7::kan* *att*Tn*7*::Tn*7*-SR1/*nilB26*-FLAG-26	NA	Kan, Erm, Str	(Bhasin et al., 2012)
HGB1211	*Xenorhabdus nematophila* Δ*nilR16::Str* Δ*SR1-7::kan* *att*Tn*7*::Tn*7*-SR1/*nilB*-FLAG26 *nilC19-M1Z* (from HGB1521)	NA	Amp, Kan, Erm, Str	This Study
HGB1808	*Xenorhabdus nematophila* Δ*nilR16::Str* Δ*SR1-7::kan* *att*Tn*7*::Tn*7*-SR1/*nilB37*-FLAG-379	NA	Amp, Kan, Erm, Str	(Bhasin et al., 2012)
HGB1207	*Xenorhabdus nematophila* Δ*nilR16::Str* Δ*SR1-7::kan* *att*Tn*7*::Tn*7*-SR1/*nilB38*-FLAG-399	NA	Amp, Kan, Erm, Str	(Bhasin et al., 2012)
HGB1681	*Xenorhabdus innexi* WT isolated from *Steinernema scapterisci* nematodes	NA	NA	(Kim et al., 2017)
HGB1699	*Xenorhabdus bovienii*-Sf-FL WT isolated from *Steinernema feltiae* Florida nematodes	NA	NA	(Murfin et al., 2015)
HGB1262	*Escherichia coli* BW29427 (CGSC#: 14194)	pURR25	Strep, Kan	(Teal et al., 2006;Sugar et al., 2012)
HGB2018	*X. nematophila* (HGB800) *att*Tn*7*::Tn*7*-GFP (from HGB1262)	NA	Cm, Amp	This Study
HGB2171	*X. innexi* (HGB 1681) *att*Tn*7*::Tn*7*-GFP (from HGB1262)	NA	Amp, Kan	(Kim et al., 2017)

HGB1865	*X. bovienii* (HGB1699) *att*Tn*7*::Tn*7*-GFP (from HGB1262)	NA	Kan	(Murfin et al., 2018)
HGB1430	*X. nematophila* (HGB007) Δ*SR1 kefA*::pJMC001-GFP (from HGB1783)	NA	Amp, Kan, Cm	(Bhasin et al., 2012)
HGB1431	*X. nematophila* (HGB007) *kefA*::pJMC001-GFP (from HGB1783)	NA	Amp, Kan, Cm	This Study
HGB1508	*X. nematophila* (HGB007) Δ*SR1 kefA*::pJMC001-GFP *att*Tn*7*::eTn*7* (from HGB1783)	NA	Amp, Kan, Cm, Erm	(Chaston et al., 2013)
HGB1509	*X. nematophila* (HGB007) Δ*SR1*, *kefA*::pJMC001-GFP *att*Tn*7*::Tn*7*-SR1 (from HGB1783)	NA	Amp, Kan, Cm, Erm	(Chaston et al., 2013)
HGB1495	Δ*nilR16::Str* Δ*SR1-7::kan* *att*Tn*7*::empty Tn*7* *kefA*::pJMC001-GFP (from HGB1783)	NA	Amp, Kan, Strep, Cm	(Bhasin et al., 2012)
HGB1496	Δ*nilR16::Str* Δ*SR1-7::kan* *att*Tn*7*::Tn*7*-SR1 *kefA*::pJMC001-GFP (from HGB1783)	NA	Amp, Kan, Strep, Cm	(Bhasin et al., 2012)

Table 2: Significantly differential proteins detected in the whole cell samples ordered by significance.

ProteinID	Student's T-test Fold Difference Δ*SR1* (HGB1495) vs. WT (HGB1496)	Student's T-test p-value Δ*SR1* (HGB1495) vs. WT (HGB1496)	Annotation via UniProt, STRING, and PaperBLAST[a]
XNC1_0327|ID:1120623|RpsQ|	9.58264	7.58E-05	30S ribosomal subunit protein S17
XNC1_2053|ID:1123413|YeiG|	3.74479	0.042141	S-formylglutathione hydrolase; formaldehyde detoxification
XNC1_3853|ID:1124183|FtsB|	3.40784	0.02679	Essential cell division protein. Septal cell wall synthesis inhibition for cell division coordination.
XNC1_4381|ID:1122482|	2.41503	0.047031	Xylanase/chitin deacetylase
XNC1_2249|ID:1123478|	1.66335	0.019203	Hypothetical protein; putative exported protein; Trypsin-like serine protease superfamily. NilR repressed.
XNC1_0005|ID:1124557|	1.23376	0.003795	Putative secreted protein
XNC1_1263|ID:1121121|CsrA|	1.22092	0.015185	Carbon storage regulator, post-translational activator of flhDC expression, regulates biofilm formation, RNA-binding A key translational regulator that binds mRNA to regulate translation initiation and/or mRNA stability. Mediates global changes in gene expression, shifting from rapid growth to stress survival by linking envelope stress, the stringent response and the catabolite repression systems

XNC1_2486|ID:1123580|	1.18659	0.043023	Putative polysaccharide deacetylase lipoprotein; UDP-glucuronate 4-epimerase
XNC1_0268|ID:1120592|ArgC|	1.05026	0.045164	N-acetyl-gamma-glutamylphosphate reductase
XNC1_2792|ID:1121766|NilC|	-10.558	3.92E-05	Internal control
XNC1_2789|ID:1123731|NilB|	-8.29132	3.97E-05	Internal control
XNC1_3140|ID:1121900|	-7.5495	0.001465	Aminoglycoside 3'-N-acetyltransferase
XNC1_3270|ID:1121939|RseC|	-6.94633	8.85E-05	Regulator of sigma E (sigma 24) factor. May play a role in reduction of the SoxR iron-sulfur cluster. Part of the rseD-rpoE-rseA-rseB-rseC operon
XNC1_4499|ID:1122518|Tag|	-6.15121	0.000211	tag 3-methyl-adenine DNA glycosylase I, constitutive
XNC1_4367|ID:1122468|	-5.59392	0.000165	*tusC* tRNA processing enzyme?
XNC1_2221|ID:1121532|TacA|	-5.37064	0.000635	TacA antitoxin
XNC1_4620|ID:1122566|PaaE|	-5.12934	0.001247	Putative phenylacetic acid degradation NADH oxidoreductase paaE 2Fe-2S, Complete proteome, Iron, Iron-sulfur, Metal-binding, Oxidoreductase
XNC1_0165|ID:1122678|MutM|	-4.86999	0.000123	Formamidopyrimidine DNA glycosylase, also acts on 5-formyluracil and 5-hydroxymethyluracil

XNC1_1032|ID:1122989|	-3.90096	0.00662	Uncharacterized, has GO: lipid metabolism; putative lipase
XNC1_3841	-3.70846	8.63E-05	Restriction modification system DNA specificity domain:Filamentation induced by cAMP protein Fic
XNC1_1003|ID:1120967|	-3.2993	0.019022	Hypothetical, catalytic activity/iron-sulfur cluster binding.
XNC1_1929|ID:1121399|	-3.24001	0.006381	Hypothetical, magnesium and manganese binding. Hydrolase activity.
XNC1_2143|ID:1123449|YecP|	-2.96338	0.047181	Putative methyltransferase with S-adenosyl-L-methionine-dependent methyltransferase domain
XNC1_4064|ID:1122336|PdxA|	-2.42829	0.000224	Pyridoxine (vitamin B6) biosynthesis 4-hydroxythreonine-4-phosphate dehydrogenase metal binding
XNC1_4231|ID:1124363|HipA|	-2.14062	0.049747	Toxin in toxin/antitoxin pair
XNC1_2535|ID:1123595|	-1.73266	0.013004	Hypothetical, intein-mediated protein splicing
XNC1_4255|ID:1124379|YjgQ|	-1.72416	0.009504	LptG; LPS transport, inner membrane protein
XNC1_3551	-1.36046	0.032782	Hypothetical protein, near sugar fermentation stimulation protein B
XNC1_2841|ID:1123750|YcdS|	-1.35837	0.029872	HmsH; PgaA; Putative outer membrane protein with transferase domain. PNAG secretion and biosynthesis.

XNC1_2818|ID:1121781|NuoI|	-1.15099	0.024956	NADH dehydrogenase I subunit L
XNC1_1277|ID:1123061|YfiA|	-1.09532	0.033991	Ribosome associated factor, stabilizes ribosomes against dissociation. NilR repressed.

[a](Jensen et al., 2009;Price and Arkin, 2017;UniProt, 2021)

Table 3: Significantly differential proteins detected in the supernatant samples ordered by significance.

ProteinID	Student's T-test Fold Difference Δ*SR1* (HGB1495) vs. WT (HGB1496)	Student's T-test p-value Δ*SR1* (HGB1495) vs. WT (HGB1496)	Annotation via UniProt, STRING, and PaperBLAST[a]
XNC1_0794|ID:1120877|AccB|	6.66447	1.54E-05	Acetyl-CoA carboxylase, biotin carboxyl carrier protein subunit
XNC1_2527|ID:1121673|	4.50272	3.76E-05	Type VI secretion system effector (Hcp1 family)
XNC1_3692|ID:1124109|Rhs|	4.48753	1.94E-05	Rhs family protein
XNC1_2986|ID:1121851|	3.93102	0.002872	Myo-inositol utilization transcriptional regulator IolR, or phosphosugar-binding transcriptional regulator, RpiR family
XNC1_0542|ID:1120757|	3.66832	0.000342	Putative toxin of the neighboring ParE antitoxin
XNC1_3741|ID:1122218|FecA|	3.27566	0.035184	Iron(III) dicitrate transport protein
XNC1_0238|ID:1120576|	2.72792	0.028309	Dihydrodipicolinate synthase
XNC1_2278|ID:1121563|	2.6597	0.009691	DNA-binding protein Cro/CI family. Possible toxin from toxin/antitoxin system.
XNC1_1779|ID:1121334|	2.20412	0.020734	Putative surface protein; some similarity to a predicted UDP-N-acetylglucosamine acyltransferase from *Myroides odoratus* NCTC11179_00821
XNC1_1708|ID:1123250|XcnD|	1.90942	0.04427	Xenocoumacin synthesis acyl-CoA dehydrogenase
XNC1_1136|ID:1121043|RecJ|	1.74231	0.02568	ssDNA exonuclease
XNC1_1449|ID:1121211|MoaC|	1.63047	0.032527	Molybdenum cofactor biosynthesis protein C; Together with MoaA, is involved in the conversion of 5'- GTP to cyclic pyranopterin monophosphate (cPMP or molybdopterin precursor Z)

XNC1_1172|ID:1123031|	1.38513	0.048399	AMP-dependent synthetase/ligase
XNC1_3157|ID:1121905|YfcG|	1.05986	0.003298	Putative glutathione S-transferase enzyme with thioredoxin-like domain
XNC1_0051|ID:1122631|	1.0226	0.016938	Inner membrane protein yjcH,
XNC1_4587|ID:1124526|	1.01995	0.03691	Hypothetical. Predicted secreted (SPI). DUF930 domain containing protein. Found in Rhizobia. NilR repressed.
XNC1_2792|ID:1121766|NilC|	-11.8249	1.03E-05	Internal control
XNC1_2789|ID:1123731|NilB|	-10.4337	1.35E-06	Internal control
XNC1_1414|ID:1121188|CydB|	-5.28305	0.000135	Cytochrome bd-I ubiquinol oxidase subunit 2
XNC1_1406|ID:1121180|SdhD|	-4.35286	0.000339	Succinate dehydrogenase, hydrophobic subunit, cytochrome b556 with SdhC; Membrane-anchoring subunit of succinate dehydrogenase
XNC1_0503|ID:1120736|RimP|	-3.96888	0.00024	Ribosome maturation factor RimP
XNC1_3812|ID:1124161|OsmY|	-3.72302	0.030845	Osmotically inducible protein Y
XNC1_3090|ID:1123880|GpA|	-3.1867	0.039814	Replication gene A protein
XNC1_0493|ID:1120729|ObgE|	-2.97453	0.035261	Putative GTP-binding protein with nucleoside triP hydrolase domain. Plays a role in the stringent response, perhaps by sequestering 50S ribosomal subunits and decreasing protein synthesis.
XNC1_3547|ID:1122102|	-2.43868	0.03406	Putative exported protein. Homolog of *Burkholderia pseudomallei* Type IV secretion system conjugative transfer protein X994_308. Linked with a possible CsrA homolog.
XNC1_2276|ID:1123486|DapA|	-2.42024	0.01801	4-hydroxy-tetrahydrodipicolinate synthase

XNC1_3032|ID:1123839|NqrF|	-2.29449	0.041516	Na(+)-translocating NADH-quinone reductase subunit F. Accepts electrons from NADH and reduces ubiquinone-1 to ubisemiquinone by a one-electron transfer pathway. Note that ubiquinone mutants have reduced glycogen storage levels.
XNC1_4107|ID:1124319|	-1.7161	0.002672	Putative capsid protein of prophage CP-933C (major head protein)
XNC1_2565|ID:1123613| YgiW/VisP BOF family protein	-1.42966	0.006652	Found to increase biofilm surface coverage when deleted in *Aggregatibacter*
XNC1_1327|ID:1123076|DacA|	-1.19536	0.004716	D-alanyl-D-alanine carboxypeptidase. Removes C-terminal D-alanyl residues from sugar-peptide cell wall precursors.

[a](Jensen et al., 2009;Price and Arkin, 2017;UniProt, 2021)

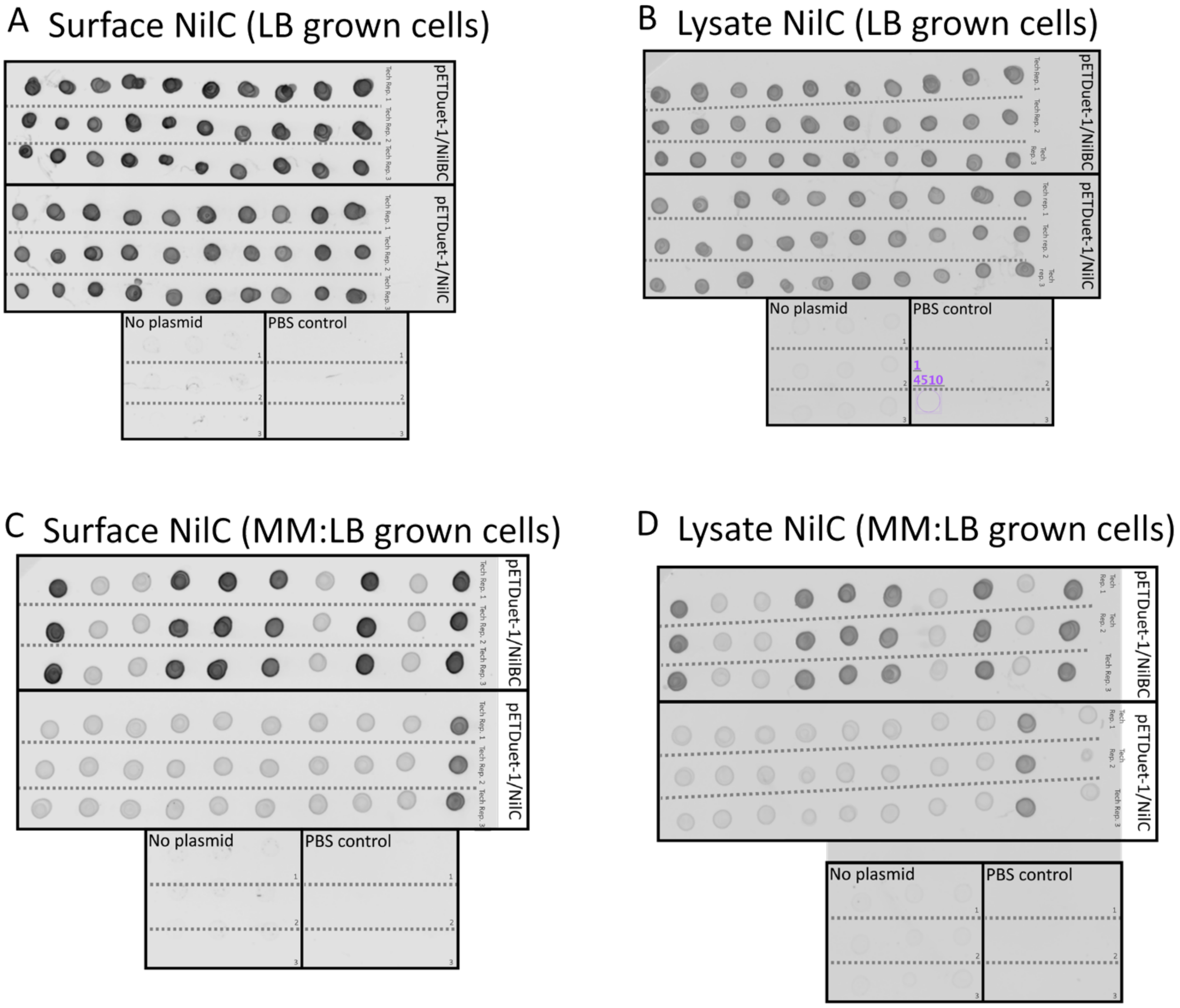

Figure 1. Immuno-dot blots of whole cells (**A,C**) or lysates (**B,D)** of *E. coli* carrying pET-Duet-1 containing P_{T7}-*nilC* and P_{T7}-*nilB* (top three rows), P_{T7}-*nilC* only (middle three rows), or no plasmid (bottom three rows, left) after growth in LB. (**A,B**) or minimal medium glucose (MM) supplemented with 1% LB (MM:LB) and 2 h of induction with IPTG.1x PBS was used as a control for background fluorescence (bottom three rows, right). Samples were spotted in technical triplicate onto nitrocellulose membranes and probed with rabbit anti-NilC primary antibody and goat anti-rabbit secondary antibody bound to a IRDye 680RD fluorophore. Emission intensity was quantitated using an Odyssey Infrared Imaging System and displayed in Figure 1. For the MM:LB treatments, after subculturing, 4/10 and 9/10 replicates of *E. coli* pET-DUET *nilB/nilC* and pET-DUET *nilC* alone, respectively, did not leave lag phase growth (measured via OD_{600}) and did not express high levels of NilC (either in lysates or on the surface).

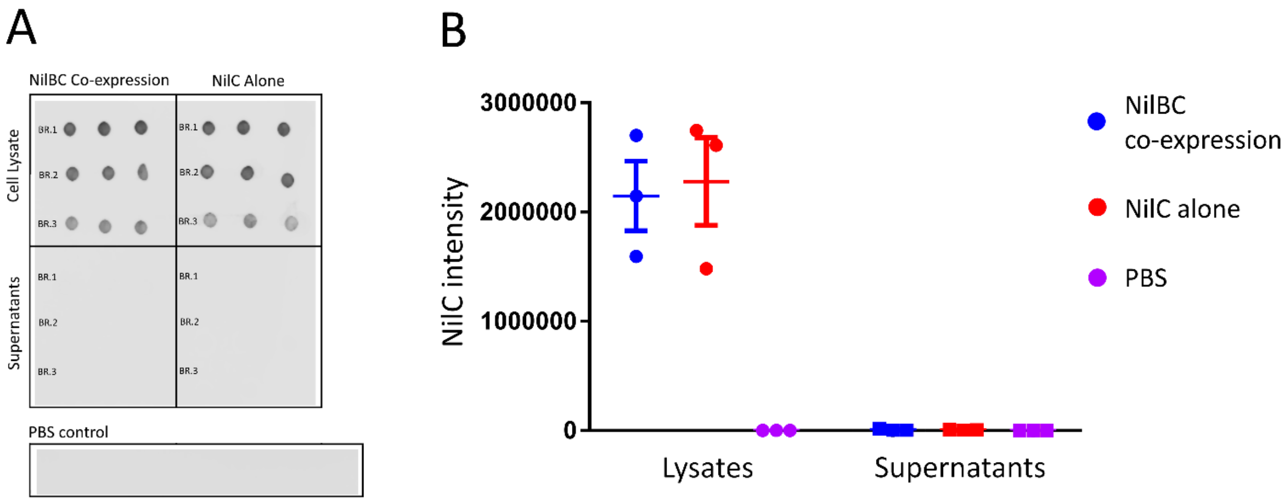

Figure 2. NilC surface exposure is not driven by cell lysis. (A) Immuno-dot blots of *E. coli* BL21 C43 co-expressing NilB and NilC or expressing NilC alone. Sterile PBS was used as a control for background fluorescent signal. Horizontal dots represent technical replication, vertical dots represent biological replication. The immunoblot was probed with anti-NilC antibody and a secondary antibody bound to a IRDye 680RD fluorophore. Emission intensity was quantitated using an Odyssey Infrared Imaging System. (B) Cellular lysate fractions for both strains demonstrate comparable expression of NilC in both strains. Error bars display the standard error of the mean. A Tukey's honestly significant difference test was performed and revealed no significant difference between NilC present in the spent media of either strain and NilC present in the sterile PBS control.

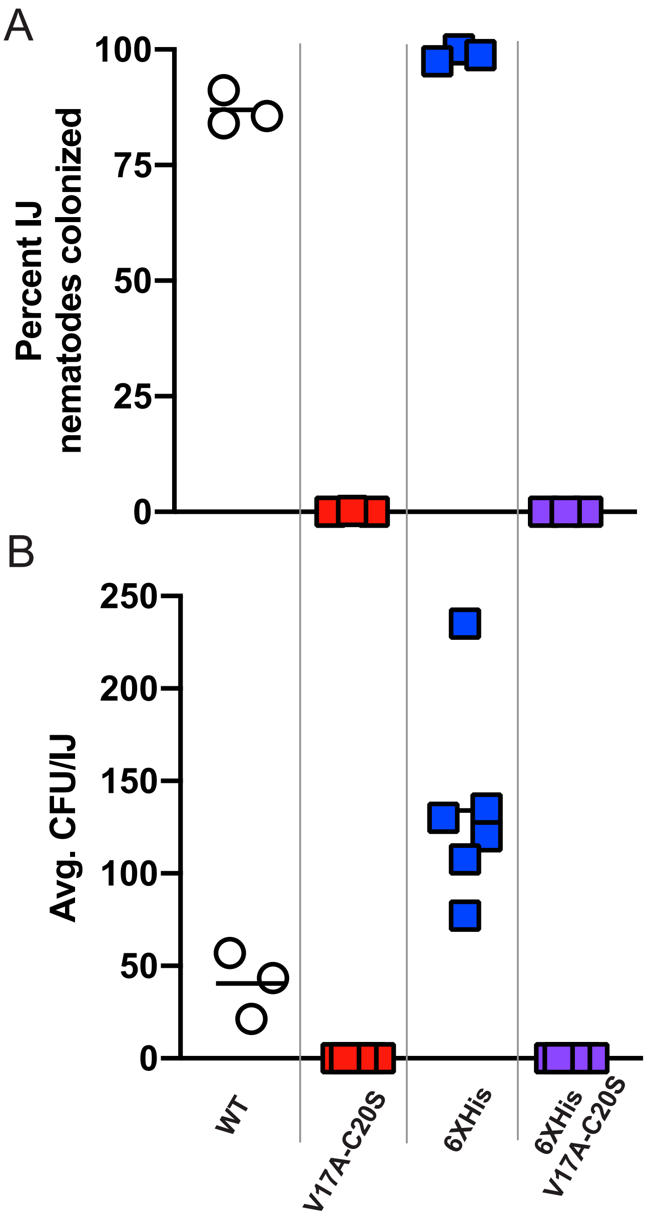

Figure 3. Histidine tag does not negatively affect NilC function in colonization whereas loss of lipidation site does. *X. nematophila* colonization of *S. carpocapsae* nematodes was measured as **A)** percent of infective juvenile (IJ) nematodes with visible green-fluorescent protein expressing *X. nematophila* within the intestinal receptacle, observed by fluorescence microscopy or **B)** the average colony forming units (CFU) per IJ as determined by surface sterilization, grinding and plating. Wild type (WT) (open circles) and *X. nematophila* Δ*SR1* carrying ectopic SR1 with *nilC* modifications (filled squares): two signal sequence amino acid changes (V17A-C20S) that eliminate the lipobox (red and purple squares), and a C-terminal 6X-His tag (6XHis) (blue and purple squares) insertion, either independently (red and blue squares) or combined (purple squares). Strains used to monitor colonization frequency expressed the green fluorescent protein from the *kefA* locus.

A

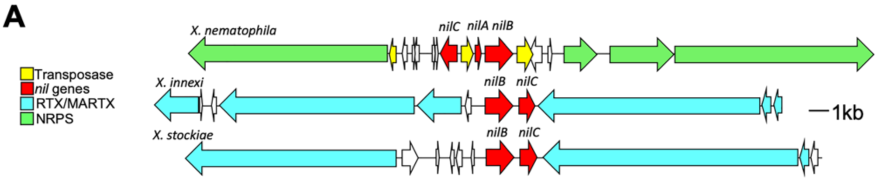

B

	XnNilC	XnC23NilC	XnF1NilC	XnwebsteriNilC	XnanatolienseNilC	XiDSMNilC	XiATCCNilC	XsDSMNilC	XsKK7NilC	XsKJ12NilC
XnNilC	0.00	0.00	0.00	0.00	0.07	0.69	0.69	0.70	0.71	0.71
XnC23NilC	0.00	0.00	0.00	0.00	0.06	0.69	0.69	0.70	0.71	0.71
XnF1NilC	0.00	0.00	0.00	0.00	0.07	0.69	0.69	0.70	0.71	0.71
XnwebsteriNilC	0.00	0.00	0.00	0.00	0.06	0.69	0.69	0.70	0.71	0.71
XnanatolienseNilC	0.07	0.06	0.07	0.06	0.00	0.69	0.69	0.71	0.72	0.71
XiDSMNilC	0.69	0.69	0.69	0.69	0.69	0.00	0.00	0.69	0.75	0.70
XiATCCNilC	0.69	0.69	0.69	0.69	0.69	0.00	0.00	0.69	0.75	0.70
XsDSMNilC	0.70	0.70	0.70	0.70	0.71	0.69	0.69	0.00	0.54	0.06
XsKK7NilC	0.71	0.71	0.71	0.71	0.72	0.75	0.75	0.54	0.00	0.54
XsKJ12NilC	0.71	0.71	0.71	0.71	0.71	0.70	0.70	0.06	0.54	0.00

C

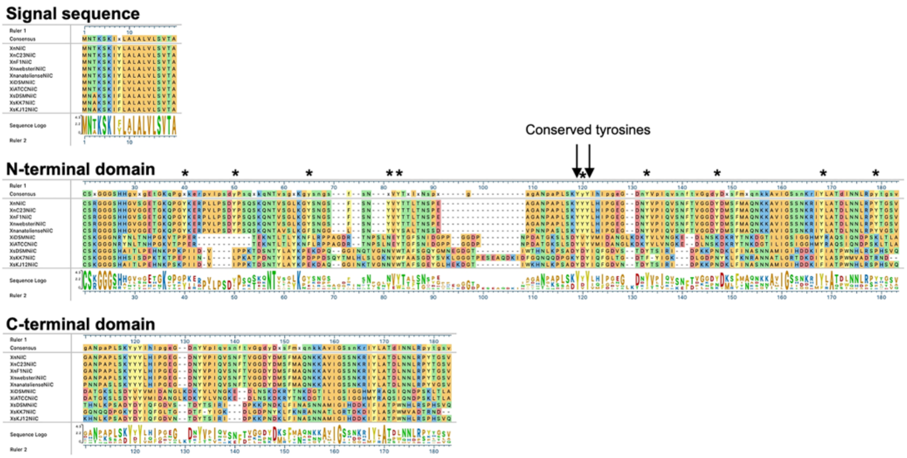

D

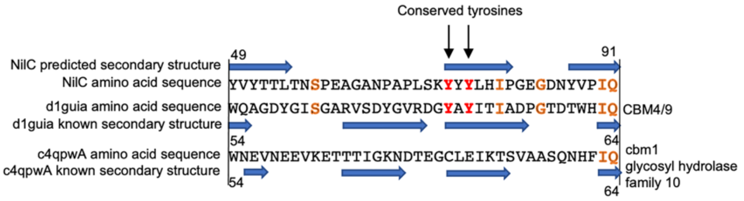

Figure 4. A) Schematic diagram of the genomic context of *nilB* and *nilC* homologs and **B)** CLUSTAL Omega uncorrected pairwise distance of and **C)** amino acid sequence alignment of NilC from strains of *X. nematophila* (ATCC19061; F1; C2-3; anatoliense, and websteri), *X. innexi* (DSM 16336 and HGB1681) and *X. stockiae* (DSM 17904, KK7.4, and KJ12). The amino acid alignment is divided according to the signal sequence and N- and C-terminal domains identified in this study. N-terminal domain tyrosines conserved among all *Xenorhabdus* NilC homologs are indicated with arrows, and those conserved among the *X. nematophila* NilC homologs are indicated by asterisks. **D)** Sequence homology alignment of segments (amino acids noted at the start and end of each segment) of NilC and d1gua, a galactose-binding domain-like, CBM4/9 family, and NilC and c4qpwa, a carbohydrate binding 1, glycosyl hydrolase domain, as detected using Phyre 2.0. The blue block arrows indicate regions of beta-sheet secondary structure, either predicted (NilC) or known (d1gua and c4qpwA).

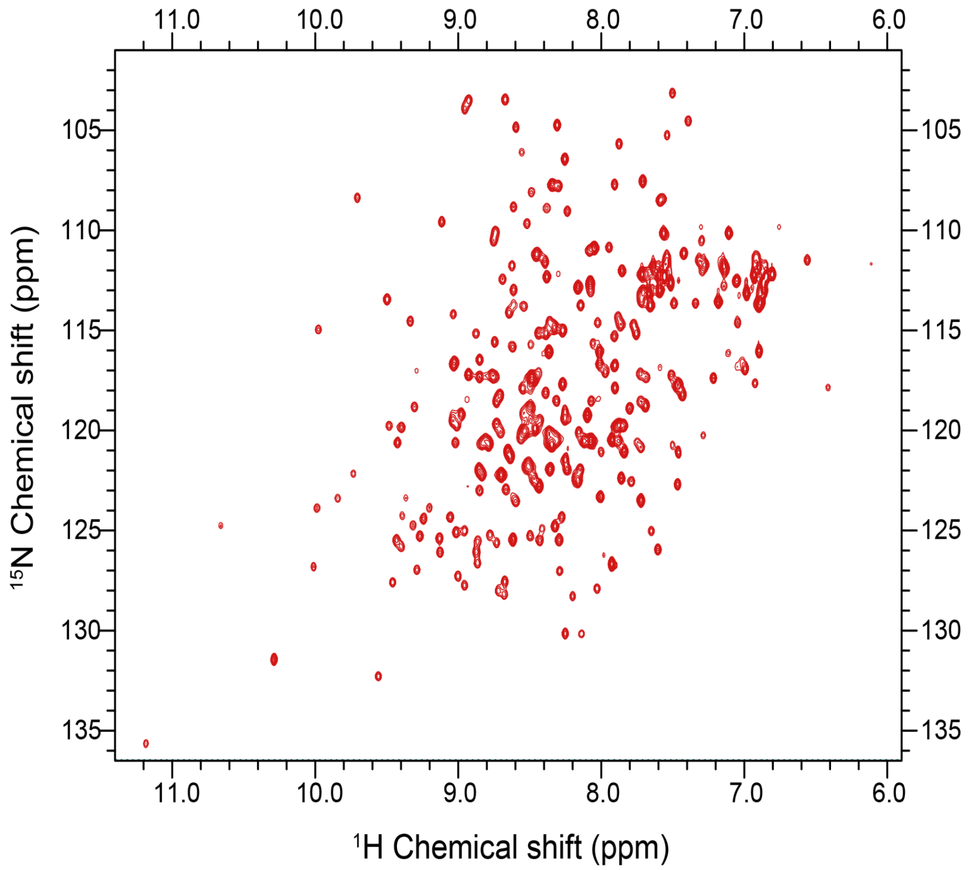

Figure 5. NilC solution NMR. 2D ^{1}H-^{15}N HSQC of ^{15}N uniformly labeled NilC was collected at 27 °C in a 750 MHz NMR spectrometer.

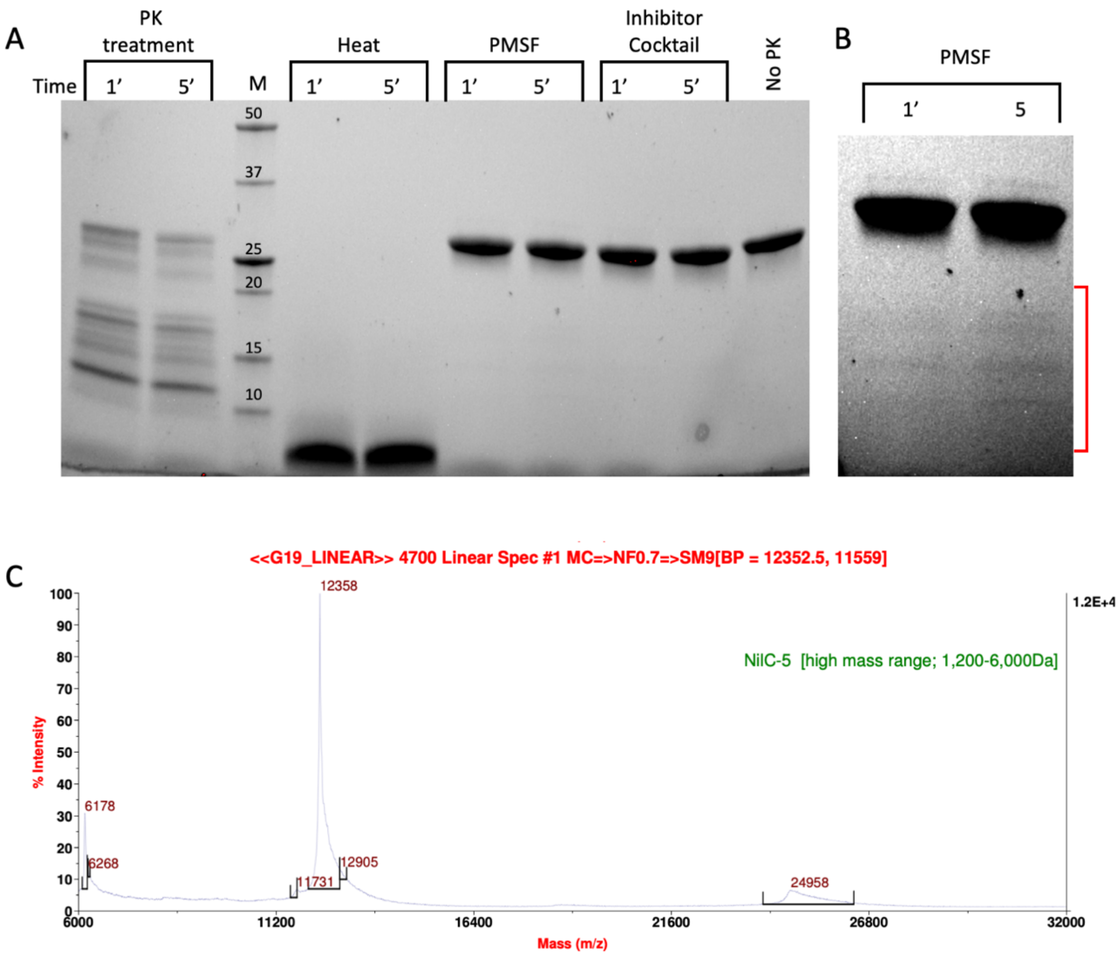

Figure 6. NilC limited proteolysis. (A) Proteinase K was used to digest purified NilC for 1 or 5 minutes, at which time samples were further subjected to no treatment, heat, or one of two protease inhibitor regimens. All samples were left at 4°C for ~1 week before running SDS-PAGE. The heat treatment accelerated Proteinase K activity and led to complete digestion, whereas PMSF or protease inhibitor cocktail prevented any further digestion. (B) Indeed, very little proteolysis had occurred after 5 minutes, as seen in this higher contrast enlarged section of the same gel. Fortuitously, however, the extended time at 4°C created the opportunity for Proteinase K to act on the SDS-treated NilC, leading to the limited digestion. (C) The mass of the major breakdown product was determined by Electrospray Ionization Mass Spectrometry run in the positive mode.

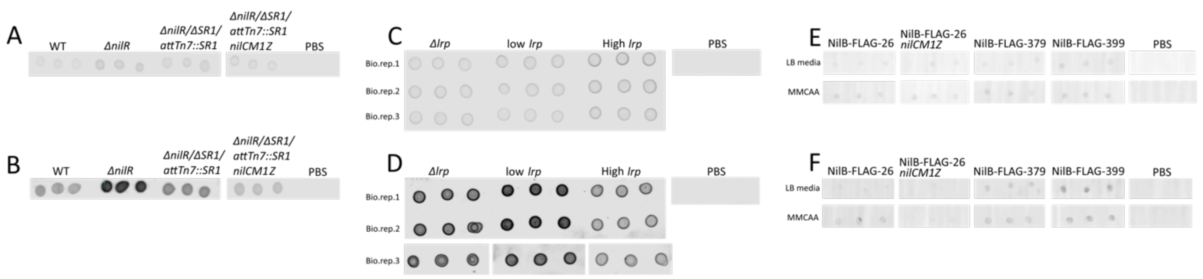

Figure 7. Immuno-dot blots of lysates (**A,C,E**) or whole cells (**B,D,F**) of *X. nematophila* preparations of *X. nematophila* wild type (WT), *ΔnilR*, *ΔnilR ΔSR1* with wild type SR1, SR1 with a *nilCM1Z* mutation (*nilCM1Z*) or *nilB*-FLAG insertions (FLAG-26, FLAG-379, FLAG-399) at the *att*Tn*7* site downstream of *glmS*, and *Δlrp* with vector only (*Δlrp*) or plasmids expressing low or high levels of Lrp were spotted onto nitrocellulose membranes. All cells were grown in LB medium except for samples shown in E and F that were grown in minimal medium with casamino acids (MMCAA; quantified data shown in Fig. S8). NilC was detected by immunoblotting with anti-NilC antibodies. 1x PBS was used as a control for background fluorescence. Samples were spotted in technical triplicate onto nitrocellulose membranes and probed with Rabbit anti-NilC primary antibody and Goat anti-rabbit secondary antibody bound to a IRDye 680RD fluorophore. Emission intensity was quantified using an Odyssey Infrared Imaging System and displayed in Fig. 3 and Fig. S8.

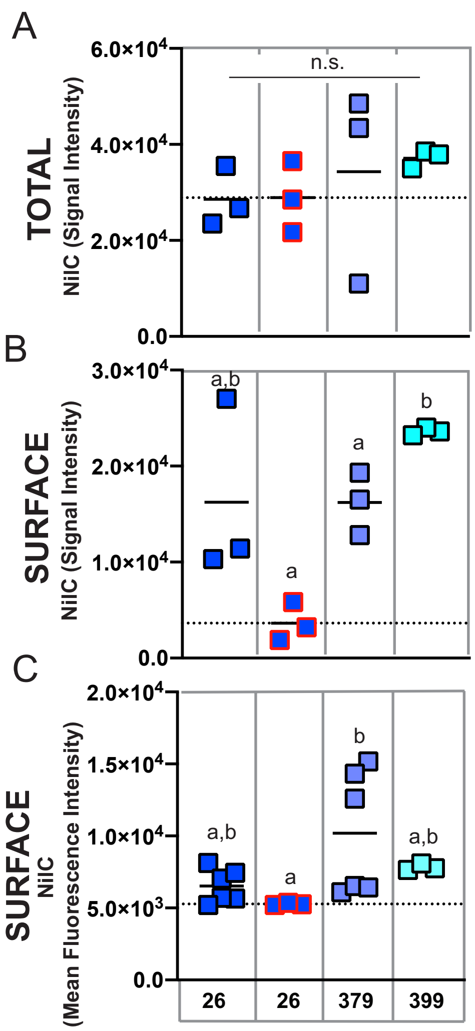

Figure 8. NilB variants influence *X. nematophila* NilC surface exposure. Cell lysate (A) or whole cell (B, C) preparations of *X. nematophila ΔnilR ΔSR1* strains grown in minimal medium supplemented with casamino acids. *ΔnilR ΔSR1* with SR1 with wild type *nilC* (black outline) or a *nilCM1Z* mutation (red outline) and *nilB*-FLAG insertions (blue shaded squares, amino acid site of insertion noted beneath) at the *att*Tn*7* site were spotted onto nitrocellulose membranes (A, B) or analyzed by flow cytometry (C). NilC protein was detected by immunoblotting with anti-NilC antibodies. Each symbol represents an individual replicate, and lines indicate mean. Significantly different groups within each panel are indicated with different letters (tested using Welch's ANOVA test with Dunnett’s T3 post-hoc multiple comparisons analysis). Dashed axis lines indicate the mean of the *nilCM1Z* datapoints.

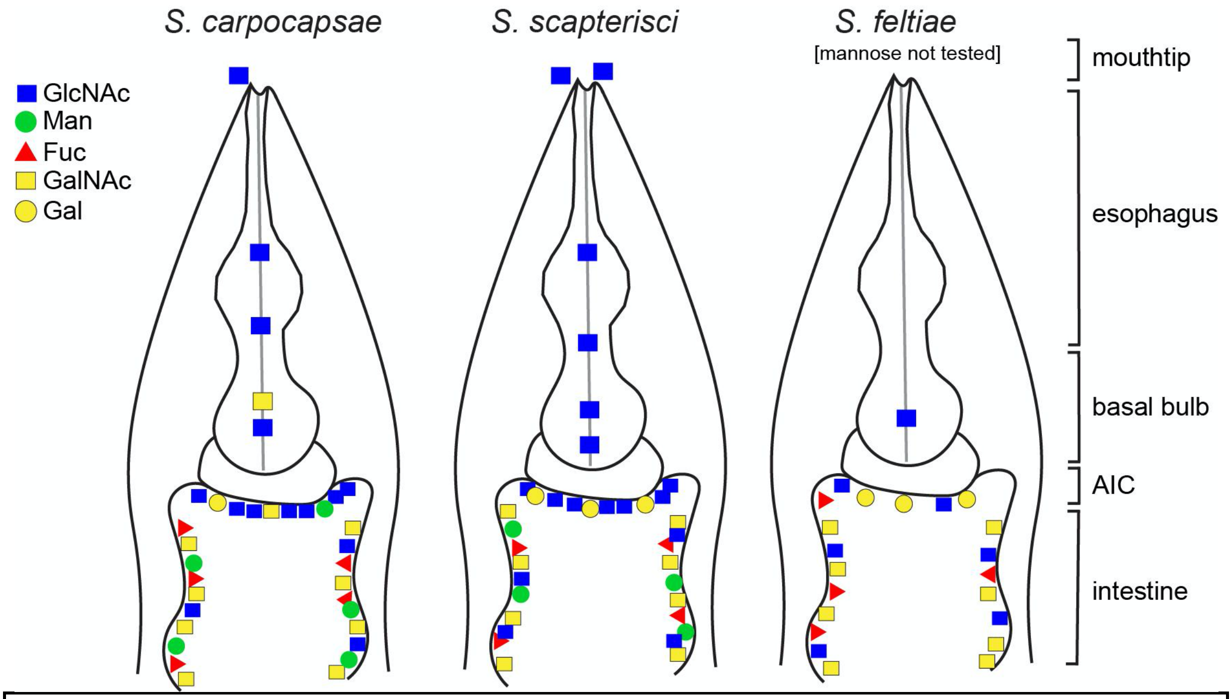

Figure 9. Summary of predicted identity and localization of glycan sugar residues in the gastrointestinal tract of *S. carpocapsae, S. scapterisci,* and *S. feltiae*. The number at each tissue location of symbols representing an individual sugar at each tissue site represents the general frequency at which the lectin corresponding to that sugar was observed as detailed in Supplementary Data File 1.

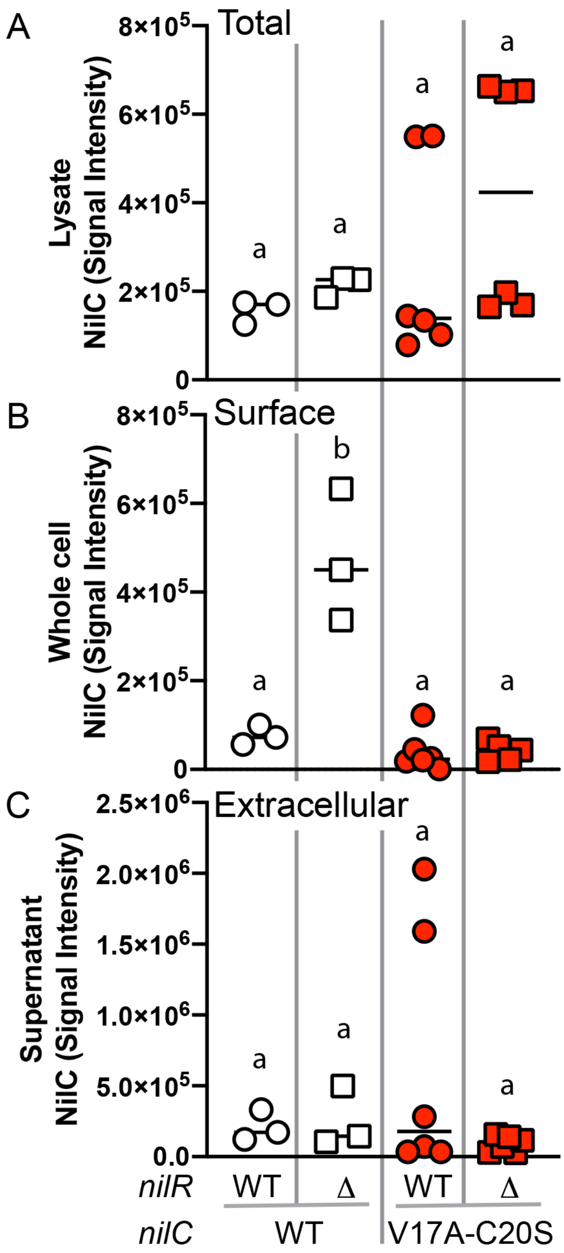

Figure 10. NilC levels in total lysed cells (A), on whole cell surfaces (B), and in cell-free supernatants (C) of *X. nematophila* carrying wild type *nilC* (white symbols) or a *nilC* lipobox mutant allele (V17A-C20S; red symbols) and grown in LB medium. The *nilC* alleles were expressed in *nilR*+ (circles) or *ΔnilR* (squares) genetic backgrounds (see methods). Significantly different groups within each panel are indicated with different letters above the bar graph (tested using one way ANOVA with Tukey's post-hoc multiple comparisons analysis). As expected, significantly more WT NilC was detected on the cell surface of *X. nematophila* cells lacking *nilR* relative to those with *nilR*. Also as expected, *X. nematophila* expressing the *nilC* lipobox mutant allele did not display NilC on the cell surface, in either the *ΔnilR* or *nilR*+ strain backgrounds. Instead, in two *nilR*+ background samples, $NilC_{V17A\text{-}C20S}$ was detected in the supernatant, suggesting that in these two samples, NilC was either secreted, or cell lysis occurred.

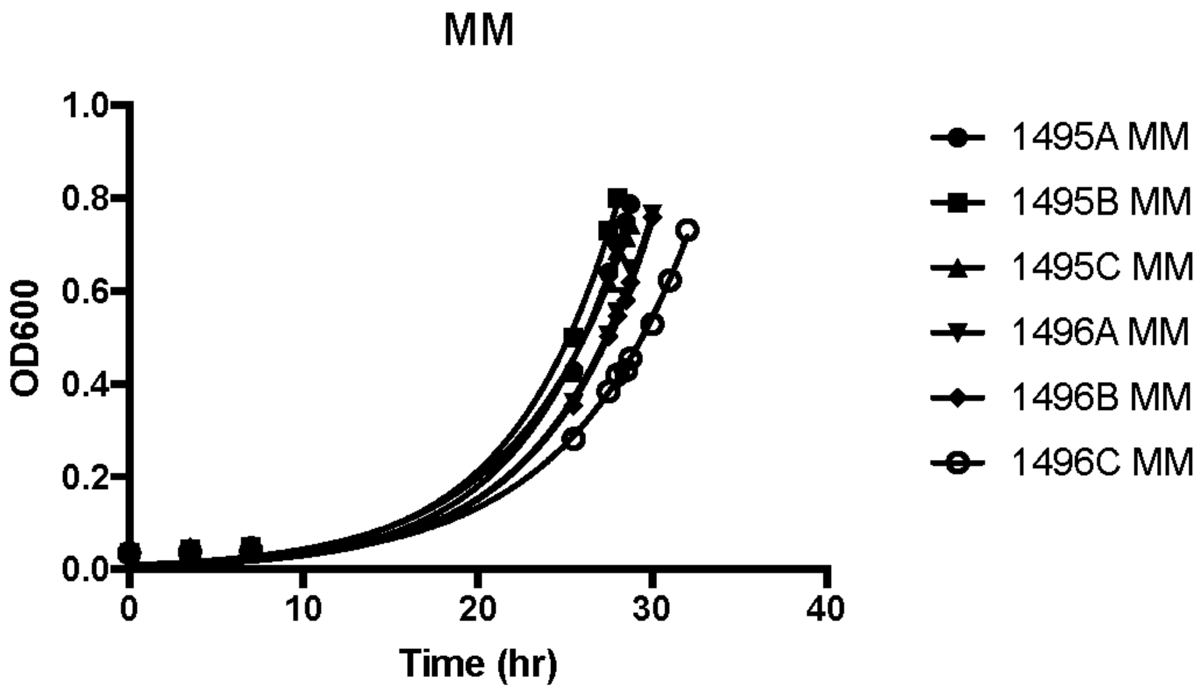

Figure 11. Growth curves of strains used for sampling to conduct metabolome and proteome analyses. Triplicate cultures (A, B, and C) of each strain (1495: *ΔSR1 att*Tn*7::*eTn*7*; 1496: *ΔSR1* attTn*7*::Tn*7/*SR1) were grown in defined medium with glucose (no casamino acids) in 500 ml flasks with 100 ml media for >24 h until OD_{600} ≅0.6 when they were harvested for proteomics and metabolomics analyses.

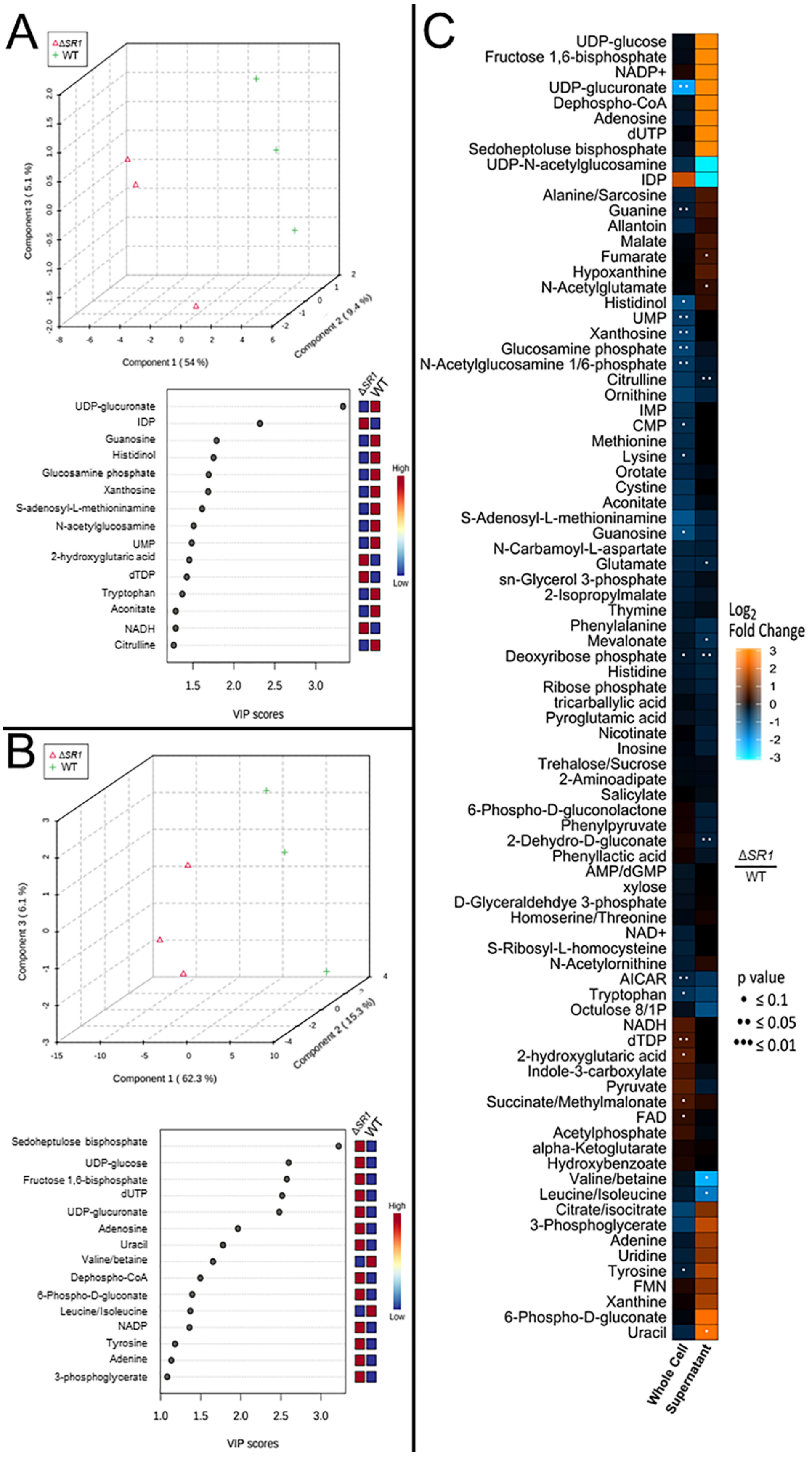
A
ΔSR1
WT
Component 1 (54 %)
Component 2 (9.4 %)
Component 3 (5.1 %)
ΔSR1
WT
UDP-glucuronate
IDP
Guanosine
Histidinol
Glucosamine phosphate
Xanthosine
S-adenosyl-L-methioninamine
N-acetylglucosamine
UMP
2-hydroxyglutaric acid
dTDP
Tryptophan
Aconitate
NADH
Citrulline
High
Low
VIP scores
B
ΔSR1
WT
Component 1 (62.3 %)
Component 2 (15.3 %)
Component 3 (6.1 %)
ΔSR1
WT
Sedoheptulose bisphosphate
UDP-glucose
Fructose 1,6-bisphosphate
dUTP
UDP-glucuronate
Adenosine
Uracil
Valine/betaine
Dephospho-CoA
6-Phospho-D-gluconate
Leucine/Isoleucine
NADP
Tyrosine
Adenine
3-phosphoglycerate
High
Low
VIP scores
C
UDP-glucose
Fructose 1,6-bisphosphate
NADP+
UDP-glucuronate
Dephospho-CoA
Adenosine
dUTP
Sedoheptoluse bisphosphate
UDP-N-acetylglucosamine
IDP
Alanine/Sarcosine
Guanine
Allantoin
Malate
Fumarate
Hypoxanthine
N-Acetylglutamate
Histidinol
UMP
Xanthosine
Glucosamine phosphate
N-Acetylglucosamine 1/6-phosphate
Citrulline
Ornithine
IMP
CMP
Methionine
Lysine
Orotate
Cystine
Aconitate
S-Adenosyl-L-methioninamine
Guanosine
N-Carbamoyl-L-aspartate
Glutamate
sn-Glycerol 3-phosphate
2-Isopropylmalate
Thymine
Phenylalanine
Mevalonate
Deoxyribose phosphate
Histidine
Ribose phosphate
tricarballylic acid
Pyroglutamic acid
Nicotinate
Inosine
Trehalose/Sucrose
2-Aminoadipate
Salicylate
6-Phospho-D-gluconolactone
Phenylpyruvate
2-Dehydro-D-gluconate
Phenyllactic acid
AMP/dGMP
xylose
D-Glyceraldehdye 3-phosphate
Homoserine/Threonine
NAD+
S-Ribosyl-L-homocysteine
N-Acetylornithine
AICAR
Tryptophan
Octulose 8/1P
NADH
dTDP
2-hydroxyglutaric acid
Indole-3-carboxylate
Pyruvate
Succinate/Methylmalonate
FAD
Acetylphosphate
alpha-Ketoglutarate
Hydroxybenzoate
Valine/betaine
Leucine/Isoleucine
Citrate/isocitrate
3-Phosphoglycerate
Adenine
Uridine
Tyrosine
FMN
Xanthine
6-Phospho-D-gluconate
Uracil
Whole Cell
Supernatant
Log2 Fold Change
ΔSR1 / WT
p value
• ≤ 0.1
•• ≤ 0.05
••• ≤ 0.01

Figure 12. Significant metabolome differences between WT and Δ*SR1* indicate TXISS role of amino acid and amino sugar metabolism. A) Whole cell metabolomics partial least squares-discriminant analysis (PLS-DA) between WT and Δ*SR1* and the associated variable of importance in projection (VIP) scores for the metabolites that contribute to the separation of metabolic profiles. B) Supernatant metabolomics PLS-DA between WT and Δ*SR1* and the associated VIP scores for the metabolites that contribute to the separation of metabolic profiles. C) Heatmap of all metabolites detected in the screen with fold change and Student's t-test p-values listed between strains for both the whole cell and supernatant analyses.

References

Bao, Y., Lies, D.P., Fu, H., and Roberts, G.P. (1991). An improved Tn*7*-based system for the single-copy insertion of cloned genes into chromosomes of gram-negative bacteria. *Gene* 109**,** 167-168.

Bhasin, A., Chaston, J.M., and Goodrich-Blair, H. (2012). Mutational analyses reveal overall topology and functional regions of NilB, a bacterial outer membrane protein required for host association in a model of animal-microbe mutualism. *Journal of Bacteriology* 194**,** 1763-1776.

Chaston, J.M., Murfin, K.E., Heath-Heckman, E.A., and Goodrich-Blair, H. (2013). Previously unrecognized stages of species-specific colonization in the mutualism between *Xenorhabdus* bacteria and *Steinernema* nematodes. *Cell. Microbiol.* 15**,** 1545-1559.

Chaston, J.M., Suen, G., Tucker, S.L., Andersen, A.W., Bhasin, A., Bode, E., Bode, H.B., Brachmann, A.O., Cowles, C.E., Cowles, K.N., Darby, C., De Leon, L., Drace, K., Du, Z., Givaudan, A., Herbert Tran, E.E., Jewell, K.A., Knack, J.J., Krasomil-Osterfeld, K.C., Kukor, R., Lanois, A., Latreille, P., Leimgruber, N.K., Lipke, C.M., Liu, R., Lu, X., Martens, E.C., Marri, P.R., Medigue, C., Menard, M.L., Miller, N.M., Morales-Soto, N., Norton, S., Ogier, J.C., Orchard, S.S., Park, D., Park, Y., Qurollo, B.A., Sugar, D.R., Richards, G.R., Rouy, Z., Slominski, B., Slominski, K., Snyder, H., Tjaden, B.C., Van Der Hoeven, R., Welch, R.D., Wheeler, C., Xiang, B., Barbazuk, B., Gaudriault, S., Goodner, B., Slater, S.C., Forst, S., Goldman, B.S., and Goodrich-Blair, H. (2011). The entomopathogenic bacterial endosymbionts *Xenorhabdus* and *Photorhabdus*: convergent lifestyles from divergent genomes. *PLoS One* 6**,** e27909.

Cowles, C.E., and Goodrich-Blair, H. (2006). *nilR* is necessary for co-ordinate repression of *Xenorhabdus nematophila* mutualism genes. *Mol. Microbiol.* 62**,** 760-771.

Hussa, E.A., Casanova-Torres, A.M., and Goodrich-Blair, H. (2015). The global transcription factor Lrp controls virulence modulation in *Xenorhabdus nematophila*. *J. Bacteriol.* 197**,** 3015-3025.

Jensen, L.J., Kuhn, M., Stark, M., Chaffron, S., Creevey, C., Muller, J., Doerks, T., Julien, P., Roth, A., Simonovic, M., Bork, P., and Von Mering, C. (2009). STRING 8--a global view on proteins and their functional interactions in 630 organisms. *Nuc. Acids Res.* 37**,** D412-416.

Kim, I.H., Aryal, S.K., Aghai, D.T., Casanova-Torres, A.M., Hillman, K., Kozuch, M.P., Mans, E.J., Mauer, T.J., Ogier, J.C., Ensign, J.C., Gaudriault, S., Goodman, W.G., Goodrich-Blair, H., and Dillman, A.R. (2017). The insect pathogenic bacterium *Xenorhabdus innexi* has attenuated virulence in multiple insect model hosts yet encodes a potent mosquitocidal toxin. *BMC Genomics* 18**,** 927.

Kwon, S.K., Kim, S.K., Lee, D.H., and Kim, J.F. (2015). Comparative genomics and experimental evolution of *Escherichia coli* BL21(DE3) strains reveal the landscape of toxicity escape from membrane protein overproduction. *Sci. Rep.* 5**,** 16076.

Murfin, K.E., Ginete, D.R., Bashey, F., and Goodrich-Blair, H. (2018). Symbiont-mediated competition: *Xenorhabdus bovienii* confer an advantage to their nematode host *Steinernema affine* by killing competitor *Steinernema feltiae*. *Environ. Microbiol.*

Murfin, K.E., Lee, M.M., Klassen, J.L., Mcdonald, B.R., Larget, B., Forst, S., Stock, S.P., Currie, C.R., and Goodrich-Blair, H. (2015). *Xenorhabdus bovienii* strain diversity impacts coevolution and symbiotic maintenance with *Steinernema* spp. nematode hosts. *mBio* 6**,** e00076.

Price, M.N., and Arkin, A.P. (2017). PaperBLAST: Text Mining Papers for Information about Homologs. *mSystems* 2.

Sugar, D.R., Murfin, K.E., Chaston, J.M., Andersen, A.W., Richards, G.R., Deleon, L., Baum, J.A., Clinton, W.P., Forst, S., Goldman, B.S., Krasomil-Osterfeld, K.C., Slater, S., Stock, S.P., and Goodrich-Blair, H. (2012). Phenotypic variation and host interactions of *Xenorhabdus bovienii* SS-2004, the entomopathogenic symbiont of *Steinernema jollieti* nematodes. *Environ. Microbiol.* 14**,** 924-939.

Teal, T.K., Lies, D.P., Wold, B.J., and Newman, D.K. (2006). Spatiometabolic stratification of *Shewanella oneidensis* biofilms. *Appl. Environ. Microbiol.* 72**,** 7324-7330.

Uniprot, C. (2021). UniProt: the universal protein knowledgebase in 2021. *Nuc. Acids Res.* 49**,** D480-D489.
